# High dietary inflammatory index associates with inflammatory proteins in plasma

**DOI:** 10.1186/s13098-024-01287-y

**Published:** 2024-02-26

**Authors:** Elisa Mattavelli, Elisa Piperni, Francesco Asnicar, Laura Redaelli, Liliana Grigore, Fabio Pellegatta, Amir Nabinejad, Sabrina Tamburini, Nicola Segata, Alberico Luigi Catapano, Andrea Baragetti

**Affiliations:** 1 Department of Pharmacological and Biomolecular Sciences “Rodolfo Paoletti”, University of Milan, Milano, Italy; 2 SISA Center for the Study of Atherosclerosis, Bassini Hospital, Cinisello Balsamo, Milan, Italy; 3 Department CIBIO, Trento University, Trento, Italy; 4 European Institute of Oncology (IEO), IRCCS, Milan, Italy; 5 IRCCS MultiMedica Hospital, Milano, Italy; 6 Department of Molecular Sciences and Nanosystems, Ca’ Foscari University, Venice, Italy

**Keywords:** Diet, Inflammation, Proteomics, Cardio-metabolic prevention

## Abstract

**Background and aim:**

Unhealthy dietary habits and highly caloric foods induce metabolic alterations and promote the development of the inflammatory consequences of obesity, insulin resistance, diabetes and cardiovascular diseases. Describing an inflammatory effect of diet is difficult to pursue, owing lacks of standardized quali-quantitative dietary assessments. The Dietary Inflammatory Index (DII) has been proposed as an estimator of the pro- or anti-inflammatory effect of nutrients and higher DII values, which indicate an increased intake of nutrients with pro-inflammatory effects, relate to an increased risk of metabolic and cardiovascular diseases and we here assessed whether they reflect biologically relevant plasmatic variations of inflammatory proteins.

**Methods:**

In this cross-sectional study, seven days dietary records from 663 subjects in primary prevention for cardiovascular diseases were analyzed to derive the intake of nutrients, foods and to calculate DII. To associate DII with the Normalized Protein eXpression (NPX), an index of abundance, of a targeted panel of 368 inflammatory biomarkers (Olink™) measured in the plasma, we divided the population by the median value of DII (1.60 (0.83–2.30)).

**Results:**

332 subjects with estimated DII over the median value reported a higher intake of saturated fats but lower intakes of poly-unsaturated fats, including omega-3 and omega-6 fats, versus subjects with estimated dietary DII below the median value (N = 331). The NPX of 61 proteins was increased in the plasma of subjects with DII > median vs. subjects with DII < median. By contrast, in the latter group, we underscored only 3 proteins with increased NPX. Only 23, out of these 64 proteins, accurately identified subjects with DII > median (Area Under the Curve = 0.601 (0.519–0.668), *p* = 0.035).

**Conclusion:**

This large-scale proteomic study supports that higher DII reflects changes in the plasmatic abundance of inflammatory proteins. Larger studies are warranted to validate.

**Supplementary Information:**

The online version contains supplementary material available at 10.1186/s13098-024-01287-y.

## Introduction

The adherence to unhealthy dietary habits and the consumption of highly caloric foods promote metabolic alterations, including obesity and insulin resistance, which are epidemic conditions leading to type 2 diabetes and cardiovascular diseases. Current guidelines constantly advise to contain the intake of calorie-dense nutrients and foods, upon the concept that reducing their metabolic burden will also constrain the inflammatory consequences of unhealthy dietary habits [1].

Anyhow, the understanding of a pro-inflammatory effect of diet, to link the intake of specific nutritional components of foods with the activation of inflammatory mechanisms, is difficult to pursue, because of shortcomings in the standardization of qualitative assessments (e.g. Food Frequency Questionnaires “FFQs”) and in the quantitative analyses of dietary consumption. Several studies tested the inflammatory potential of dietary patterns of surrogate indices of the quality of diet [2–6], although the nature of the dietary information was qualitative and different panels of biomarkers were interrogated. Furthermore, the type of assays used differed among studies and only a limited number of biomarkers related to inflammation was tested. The Dietary Inflammatory Index (DII) is a validated score [7], generally calculated from the analysis of FFQs, that has been associated with the presence or the occurrence of cardio-metabolic alterations [8–12] and cardiovascular diseases [13–16] in epidemiological studies [17]. DII normalizes the intake of each nutrient present in the foods consumed over the period of the dietary assessment for a correction factor (the “inflammatory effect score” [18]). This factor can be either positive, for nutrients that are expected to exert pro-inflammatory effects (e.g., saturated fats, to which the highest score is addressed), or negative, for nutrients that are expected to exert anti-inflammatory effects based on experimental evidence from literature (e.g. fiber, to which the lowest score is addressed) [18].

Sparse data indicate that a positive or a negative change in DII can reflect a respective biologically relevant increase or reduction in the plasma levels of some inflammatory proteins. Indeed, some data indicate that high DII relates to increased plasma levels of C-Reactive Protein (CRP) [8, 13, 19, 20], while others do not support this relation [21, 22] or failed to find an association with other common markers of inflammation [23]. Also, the association between high DII, increased blood levels of immune cells and increased levels of few other interleukins and factors (e.g. IL-1 α and TGF- β) has been only recently evaluated in marginalized populations [24, 25] or in comorbid patients [26].

Thereby, to better elucidate the relation between higher DII and inflammatory markers, we conducted a plasma proteomic study, measuring the plasmatic abundance of 368 proteins, that we previously associated with increased cardiovascular risk in independent cohorts [27, 28]. By harnessing Proximity Extension Assay (PEA; Olink™), a technology that combines the use of antibodies with unique oligonucleotides to run DNA amplification steps, we simultaneously measured the relative expression (as Normalized Protein eXpression, “NPX” [29]) of each protein, achieving an elevated degree of sensitivity to reach up to ng-pg/ml concentration ranges. Two independent studies, measuring a smaller number of proteins with this technique, found an association between higher DII and some inflammatory proteins [6, 30], and we now tested whether, enlarging the spectrum of the array, we can discover additional fingerprints of an inflammatory potential of diet.

## Materials and methods

### Study design and population

The “PLIC” (Progressione delle Lesioni Intimali Carotidee) Study was developed and followed at the Center for the Study of Atherosclerosis at E. Bassini Hospital (Cinisello Balsamo, Milan, Italy). 2.606 participants were initially included in the PLIC study from 2001 to 2003 [28, 31–33] and all the information needed for the purpose of this study was available on 663 subjects. Supplemental Fig. 1 reports the flow-chart of the study. Further information about ethic statements, inclusion criteria, sample selection, sample size statistical analysis, and selection bias are reported in Supplemental Material. This work is a cross-sectional study, and it was conducted following the standards of the STrengthening the Reporting of OBservational studies in Epidemiology (STROBE) initiative [34].

### Measurement of biochemical and clinical parameters

Blood samples were collected from antecubital vein after 12 h fasting on NaEDTA tubes (BD Vacuette) and then, centrifuged at 3,000 rpm for 12 min (Eppendorf 580r, Eppendorf, Hamburg, Germany) for biochemical parameters profiling including total cholesterol, HDL-C, triglycerides, Apolipoprotein B (ApoB), Apolipoprotein A-I (ApoA-I), glucose and C-Reactive Protein. Measurements were performed using immuno-turbidimetric and enzymatic methods through automatic analyzers (Randox, Crumlin, UK). LDL-C was derived from the Friedewald formula.

Data on pathological and pharmacological history (including lipid-lowering, glucose-lowering, anti-hypertensive, and antiplatelet therapy). Clinical and anthropometrical measures (systolic and diastolic blood pressure, Body Mass Index (BMI), waist and hips circumferences, height, and weight) and lifestyle habits as described elsewhere [32].

### Analysis of the seven days dietary records and definition of food groups, items and sub-groups

The intake of calories and macro-/micro-nutrients were analyzed from the foods that were self-reported to be consumed by the subjects in the seven-day dietary records, as previously published [32]. In brief, subjects were asked to fill in a paper version of the seven-day dietary record, at the moment of having their daily meals, with a detailed description about the type of each food consumed (e.g., type of milk consumed, if either goat milk, full fat-cow milk, semi-skimmed cow-milk), the weighted amount and the home size (e.g., number of mugs, spoons, number of portion sizes commercially available). These data were then analyzed by trained dietitians and nutritionists during the clinical evaluation of the subject, following the Guidelines dedicated for the Italian population regarding the standard portion sizes (LARN “Livelli di Assunzione di Riferimento di Nutrienti ed energia” [35] and Italian Dietary Guidelines [36]). The subjects were asked to provide more information regarding the consumed recipes, to distinguish the amount and type of the ingredients. Then, the caloric and the content of macro-/micro-nutrients in each food was estimated by interrogating the *in silico* publicly available dataset of the Food Composition Database for Epidemiological Studies in Italy (BDA) [37], which provides the information regarding the caloric and the nutritional composition of 978 foods and classifies them into “food groups”, “food subgroups” and “food items” (for instance, “*oils/butter/margarine*” are reported in BDA dataset as “food groups”, they can include “*oils and vegetable fats*” as “food subgroups”, which, as a consequence, they include “*olive oil*” as “food item”). We also consulted the available literature to detail in depth the foods that were eventually not described in the BDA dataset [38–42]. In case the dataset lacks information regarding the nutritional composition of a food or an ingredient, an alternative food with an analogous nutritional content was considered [43].

### Calculation of the DII

The intake of macro-and micro-nutrients derived from the analysis of the seven-days dietary records was employed to calculate the DII, following the algorithm proposed by Shivappa N et al. [18]. Briefly, the dietary intake estimates for each participant were converted to centered percentiles for each component referring to regionally representative global database by computing a z-score; the centered percentile was then multiplied by the corresponding “inflammatory effect scores” of each nutrient (between − 1 to + 1, when negative values indicate an anti-inflammatory effect and positive values indicate a pro-inflammatory effect). The inflammatory effect score of a food pattern resulted from the sum of the inflammatory effect scores of the nutrients included in that food pattern.

### Proteomics analysis

Proteins were measured by Proximity Extension Assay (PEA) strategy and the complete list of the proteins that are included in the Cardiovascular II, Cardiovascular III, Cardiometabolic and Inflammation panels of the Olink™ platform have been previously indicated [27]. Further methodological details are reported as Supplemental Material.

### Statistics

The statistical analyses were performed using the SPSS software (version 28.0) for Windows. Graphs were prepared using GraphPad Prism (version 8).

Linear data are presented as mean with standard deviation or as median (interquartile ranges) after verifying for normal distribution (Kolmogrov-Smirnov test). The comparison within each group was performed with simple t-test (if linear distribution) or Mann-Whitney U-test (if not-normal distribution). The variations in the expression of plasma proteins between groups of subjects were analyzed by calculating the fold changes (on log_2_ scale).

To validate the biological relevance of the DII, we built a binary outcome prediction (DII > median cohort vs. DII < median cohort) model with XGboost algorithm.

### Gradient boosting machine learning (ML) model

The model included all the significantly different proteins measured among those with DII > median vs. DII < median. The total sample was split randomly into a train set (60% of the entire cohort) and a test set (40% of the entire cohort). The XGBoost classifier model was trained in the train set with 1000 iteration rounds and < 0.001 learning rate. Hyperparameter optimization was performed by k-fold iteration internal to the training set. The most important proteins found in the optimized model were then listed by relative importance in the Random Forest classifier plot. Then we assessed the predicting performance of the algorithm in the test set by Receiver Operating Characteristic (ROC) analysis. Models were built in Python 6.4.5 with pandas, scikit–learn, NumPy, XGboost.

### Gene Ontology (GO) and KEGG pathway enrichment analysis

We conducted an enrichment analysis of biological processes with the proteins that emerged as significantly associated with higher DII, as previously published [44, 45]. The DAVID (The Database for Annotation, Visualization, and Integrated Discovery, NIAID, North Bethesda, MD, USA) platform was used for gene ontology (GO) enrichment analyses. The significant GO biological processes (GO_bp) were selected for FDR < 0.05. Then, for each GO biological process (GO_bp) we annotated the fold of enrichment, an index of the percentage of proteins belonging to a pathway, and the false discovery rate (FDR) to indicate how likely the enrichment is by chance (FDR < 0.05 indicates a statistically significant enrichment of proteins in that pathway).

## Results

### Specific food patterns and nutritional profiles from habitual diets characterize higher DII

663 subjects were asked to self-report their dietary habits in a seven-day dietary record. The clinical characteristics of the population are reported in Table 1 and the dietary data, including the amounts of food patterns consumed, the percentages of the energy deriving from the main macronutrients (%En/day), and the absolute intakes of the micro-nutrients present in the consumed food patterns (either as milligrams/day (mg/day) or micrograms/day (µg/day)) are reported in Tables 2 and 3.

**Table 1:** Clinical characteristics of the population divided by median DII. The table reports the clinical characteristics and the biochemical parameters of the population divided according to the median value of DII. N = 331 subjects displayed DII below the median (DII < median) and 332 subjects displayed DII over the median (DII > median)

	Total sample (n = 663)	DII < median (n = 331)	DII > median (n = 332)	
	*Median (25th-75th percentiles)*	*Median (25th-75th percentiles)*	*Median (25th-75th percentiles)*	*p*
**Dietary Inflammatory Index**	1.60 (0.83–2.3)	0.83 (0.29–1.18)	2.30 (1.97–2.73)	-
**Age (years)**	56 (50–61)	56 (50–61)	55 (50–60)	0.187
**Female, n (%)**	447 (67.42)	215 (64.95)	232 (69.88)	0.176
**Smokers, n (%)**	126 (19)	52 (15.71)	74 (22.29)	0.031
**Physically active, n (%)**	295 (44.49)	178 (53.78)	117 (35.24)	< 0.001
**Body Mass Index (kg/m^2)**	26.22 (23.81–28.81)	25.84 (23.5-28.23)	26.66 (24.23–29.18)	0.006
**Waist to Hips ratio**	0.8 (0-0.84)	0.79 (0.71–0.83)	0.8 (0-0.85)	0.360
**Systolic Blood Pressure (mmHg)**	130 (120–140)	130 (120–140)	130 (120–140)	0.555
**Diastolic Blood Pressure (mmHg)**	80 (80–90)	80 (80–90)	80 (80–90)	0.822
**Anti-hypertensive therapies, n (%)**	153 (23.08)	75 (22.66)	78 (23.49)	0.799
**Fasting glucose (mg/dL)**	88 (82–96)	87 (81–96)	89 (82–96)	0.347
**Glucose-lowering therapies, n (%)**	4 (0.6)	2 (0.6)	2 (0.6)	0.998
**Cholesterol (mg/dL), mean ± SD**	222.43 ± 38.55	224.07 ± 37.21	220.8 ± 39.84	0.275
**HDL-C (mg/dL), mean ± SD**	54 (45–66)	54 (45–67)	54 (46–65)	0.434
**Triglycerides (mg/dL)**	91 (64–132)	89 (63–127)	92.5 (68-137.5)	0.147
**LDL-C (mg/dL)**	144.71 ± 35.76	146.48 ± 33.88	142.92 ± 37.53	0.202
**Remnant cholesterol (mg/dL)**	18.2 (12.8–26.4)	17.8 (12.6–25.4)	24.64 ± 26.14	0.147
**Apo A1 (mg/dL)**	149.95 ± 24.58	148.99 ± 23.77	149 (132–169)	0.386
**Apo B (mg/dL), mean ± SD**	112 (96–131)	114.7 ± 24.96	112.22 ± 25.23	0.274
**Apo A1/Apo B ratio**	0.75 (0.62–0.93)	0.75 (0.63–0.95)	0.74 (0.6–0.91)	0.185
**Lipid-lowering therapies, n (%)**	56 (8.45)	27 (8.16)	29 (8.73)	0.789
**CRP (mg/L)**	0.09 (0.05–0.16)	0.08 (0.04–0.15)	0.1 (0.06–0.17)	0.004
**Previous CVD events, n (%)**	0 (0)	0 (0)	0 (0)	.
**Antiplatelet therapies, n (%)**	13 (1.96)	9 (2.72)	4 (1.2)	0.160

**Table 2:** Intakes of the food groups, subgroups and items reported to be consumed by the subjects that were divided according to median DII. The table lists the amounts of the foods groups (in bold), of the food subgroups and of the food items (in italic) consumed by the subjects with DII below the median (DII < median, N = 331) versus the subjects with DII over median (DII > median, N = 332)

	Total sample (n = 663)	DII < median (n = 331)	DII > median (n = 332)	
	*Median (25th-75th percentiles)*	*Median (25th-75th percentiles)*	*Median (25th-75th percentiles)*	*p*
**Tubers, potatoes, starch (g/day)**	**21.43 (6.89-40)**	**24.07 (7.14–44.84)**	**19.01 (5.63–35.35)**	**0.019**
**Vegetables, mushrooms (g/day)**	**200.21 (147.14-254.69)**	**234.57 (191.04-295.65)**	**164.14 (117.87-209.18)**	**< 0.001**
Tomatoes (g/day)	14.29 (0-38.58)	14.29 (0-42.86)	14.29 (0-35.36)	0.039
Dark-yellow vegetables (g/day)	7.41 (2.12-20)	13.57 (4.17–28.57)	4.29 (0.82–11.43)	< 0.001
Leafy vegetables (including salads) (g/day)	46.16 (25.69–71.43)	57.14 (34.29–82.44)	37.14 (19.47–57.14)	< 0.001
Cruciferous vegetables (g/day)	0 (0-14.29)	4.29 (0-22.5)	0 (0-8.68)	< 0.001
Other vegetables (g/day)	109.87 (71.71-148.57)	128.06 (91.43-171.72)	85.71 (58.03-125.78)	< 0.001
**Legumes and soy products (g/day)**	**12.86 (0-28.57)**	**14.29 (3.17–33.33)**	**5.7 (0-21.43)**	**< 0.001**
**Fresh fruits, dried fruits, flours, juices (g/day)**	**261.07 (168.78-350.07)**	**307.14 (238.57-411.43)**	**214.29 (119.86-301.35)**	**< 0.001**
Fresh fruit and berries (g/day)	238.21 (142.14-323.06)	285.71 (207.14–370)	190.99 (89.29-278.57)	< 0.001
Dried fruit and seeds (g/day)	0 (0-2.86)	0.71 (0-4.29)	0 (0-1.79)	< 0.001
Fruit juices and drinks (g/day)	0 (0-28.57)	0.71 (0-31.25)	0 (0-4.23)	< 0.001
**Milk and yogurt (g/day)**	**122.85 (28.12–170)**	**125 (27.86-187.86)**	**110.41 (27.61-162.41)**	**0.061**
**Cheeses (g/day)**	**34.15 (21.43–47.47)**	**33.85 (20.71–47.81)**	**34.27 (23.03–47.5)**	**0.348**
Low-fat cheeses (< 25% fat content) (g/day)	12.86 (0-22.86)	10 (0-21.43)	14.29 (0-23.42)	0.083
Other cheeses (g/day)	18.57 (9.92-30)	18.57 (9.38–30.9)	18.57 (10–30)	0.966
**Cereals, flour, pasta, bread, crackers, rusks (g/day)**	**162.33 (122-207.5)**	**174.8 (131.67-218.57)**	**154.29 (113.1-197.57)**	**< 0.001**
Refined cereals, flour, pasta, bread, crackers, rusks (g/day)	157.86 (115.71-202.68)	164.29 (126.31-208.63)	147.67 (106.07-194.67)	0.001
Whole cereals, flour, pasta, bread, crackers, rusks (g/day)	0 (0–0)	0 (0-4.29)	0 (0–0)	< 0.001
**Eggs (g/day)**	**8.33 (1.75–14.37)**	**8.57 (2.14–16.43)**	**7.41 (1.53–14.29)**	**0.240**
**Meat and meat products (g/day)**	**93.57 (67.48-123.96)**	**96 (69.71-125.64)**	**88.93 (65.46-121.18)**	**0.142**
Processed meat (g/day)	24.43 (13.53–37.4)	25.71 (13.57–37.23)	23.09 (13.42–37.71)	0.460
Meat and offal (g/day)	67.78 (44.29–91.34)	68.57 (48.95–93.01)	64.29 (42.86–89.29)	0.112
*Offal (g/day)*	0 (0–0)	0 (0–0)	0 (0–0)	0.307
*Red meat (g/day)*	35.71 (17.14–57.14)	36.54 (17.14-60)	34.01 (17.32–55.98)	0.287
*Other meat (g/day)*	22.5 (14.29–42.86)	24.29 (14.29–42.86)	21.43 (8.7-42.86)	0.339
**Fish, shellfish, mollusks (g/day)**	**28.57 (14.29-50)**	**35.71 (20.71-60)**	**25.36 (7.14–41.79)**	**< 0.001**
Oily fish (g/day)	0 (0-14.29)	0 (0–15)	0 (0-7.5)	0.002
Other fishes (g/day)	21 (7.14–36.43)	25 (10-45.61)	14.52 (0-31.27)	< 0.001
Shellfish and mollusks (g/day)	0 (0–0)	0 (0–0)	0 (0–0)	0.137
**Oils, margarins, butter, cream (g/day)**	**27.86 (20.69–35.71)**	**30 (23.04–38.35)**	**26.24 (18.81–32.63)**	**< 0.001**
Margarine (g/day)	0 (0–0)	0 (0–0)	0 (0–0)	0.771
Oils and vegetable fats (g/day)	22.86 (17.14–29.14)	25.17 (19.06–32.33)	20.71 (14.58–25.96)	< 0.001
*Olive oil (g/day)*	21.43 (15.71–27.62)	24.03 (17.86–31.43)	19.24 (14.18–24.62)	< 0.001
*Other vegetables oils (g/day)*	0 (0-1.43)	0 (0-1.74)	0 (0-0.82)	0.120
Butter and animal fats (excluding cream) (g/day)	2.86 (0-6.43)	2.86 (0-6.43)	2.85 (0-6.31)	0.888
Cream (g/day)	0 (0–0)	0 (0–0)	0 (0–0)	0.950
**Sweets, sugar, jams, ice-creams (g/day)**	**20.32 (9.66–37.39)**	**21.43 (10.31–41.43)**	**19.29 (9.29–34.86)**	**0.186**
Chocolate and cocoa (g/day)	0 (0–3)	0 (0-3.69)	0 (0-2.93)	0.938
Croissants, cookies, puddings, cakes (g/day)	35.71 (20-59.55)	39.29 (20.29–61.43)	33.47 (19.88–55.71)	0.088
**Beverages (mL/day)**	**247.94 (131.67–376.8)**	**274.61 (149.29-388.57)**	**211.92 (117.66-356.71)**	**0.002**
Non-alcoholic beverages (mL/day)	114.29 (67.32-198.12)	115 (68.57-192.86)	107.86 (64.29–200)	0.507
*Sugar-sweetened beverages* *(mL/day)*	0 (0-17.86)	0 (0-10.5)	0 (0-25.54)	0.492
*Tea (mL/day)*	0 (0-64.29)	0 (0-64.29)	0 (0-64.29)	0.503
*Coffee (mL/day)*	63.5 (38.57-90)	64.29 (38.57-90)	61.9 (38.57–85.71)	0.375
*Herbal teas, infusions (g/day)*	0 (0–0)	0 (0–0)	0 (0–0)	0.396
Alcoholic beverages (mL/day)	71.43 (10.01-202.27)	105.98 (20.47-226.21)	50 (3.39-175.28)	< 0.001
*Wine and sparkling wines* *(mL/day)*	47.2 (4.29-160.71)	85.71 (8.57-196.43)	32.73 (2.06-113.39)	< 0.001
*Beer (mL/day)*	0 (0-24.29)	0 (0-21.43)	0 (0-28.57)	0.953
*Distillates, sweets liquors, high* *alcohol beverages and high* *alcohol bitter liqueur (mL/day)*	0 (0–0)	0 (0–0)	0 (0–0)	0.744
**Aromatic herbs and spices (g/day)**	**15.2 (4.05–36.53)**	**15.29 (4.64–35.71)**	**15.09 (3.01–37.14)**	**0.410**

The nutritional composition of the consumed food patterns was then used to calculate the DII, which was 1.60 on average in the population (0.83–2.30) and, to explore which foods and nutrients mostly reflect higher DII values, we compared the nutritional and dietary profiles of the subjects with DII > median (n = 332, DII = 2.30 (1.97–2.73)) versus those of the subjects with DII < median (n = 331, DII = 0.83 (0.29–1.18)). The subjects with DII > median reported to consume not only less vegetables (including tomatoes, dark-yellow/leafy/cruciferous vegetables), legumes, and fruits (including fresh and dried fruits, flours and juices), but also less daily amount of tubers and potatoes, cereals, flour, pasta, bread, crackers and rusks (both refined and whole), oily and non-oily fishes), olive oil and wine, compared to subjects with DII < median. By contrast, the consumption of other food patterns, including milk and yogurt, cheese (including low-fat cheese), meat and meat products (including preserved, red, and white meat), shellfish and mollusks, butter, chocolate, croissant, cookies, puddings, cakes, non-alcoholic beverages (including sugar-sweetened beverages, tea and coffee), beer and spirits were comparable between the two groups (Table 2 and Supplemental Table 1).

Of note, the differences in the food patterns consumed resulted in lower daily caloric intake in subjects with DII > median compared to subjects DII < median (1592.03 (1344.00-1848.50) Kcal/die vs. 1882.61 (1587.64-2168.17) Kcal/die respectively, *p* < 0.001; Table 3). In search of a possible explanation for this finding, we profiled the percentages of energy deriving from the principal caloric-yielding components of diet, which are the macronutrients. Yet, the percentages of energy from carbohydrates (including soluble carbohydrates) and proteins were comparable between subjects with DII > median vs. subjects with DII < median (49.48 ± 6.36% vs. 50.00 ± 6.27% from carbohydrates, *p* = 0.284; 15.84 (13.29–18.96)% vs. 17.21 (14.76–20.08)% from soluble carbohydrates, *p* < 0.001; 16.04 (14.73–17.66)% vs. 16.04 (14.65–17.59)%, *p* = 0.553 from proteins (Table 3)). By contrast, although the energy deriving from the intakes of total lipids, and of monounsaturated fats (MUFA) were comparable between both groups (for total lipids: 35.49 ± 5.68% in subjects with DII > median vs. 34.73 ± 5.53% in subjects with DII < median, *p* = 0.083; for MUFA 16.26 (14.22–17.93)% in subjects with DII > median vs. 16.25 (14-31-18.33)% in subjects with DII < median, *p* = 0.647 (Table 3)), subjects with DII > median reported to acquire higher energy deriving from the dietary intake of saturated fats, but reduced energy deriving from the intake of polyunsaturated fats (PUFA) versus subjects with DII < median (for saturated fats: 12.05 ± 2.55% vs. 11.08 ± 2.36% respectively, *p* < 0.001 (Table 3)); for PUFA: 3.88 (3.39–4.60)% vs. 4.12 (3.55–4.90)%, respectively *p* = 0.002 (Table 3)). Also, the percentages of energy deriving from the intakes of omega-3 PUFA and omega-6 PUFA were reduced in subjects with DII > median vs. subjects with DII < median (for omega-3: 0.57 (0.49–0.69)% vs. 0.64 (0.53–0.77)% respectively, *p* < 0.001; for omega-6 3.24 (2.73–3.88)% vs. 3.42 (2.88–4.12)% respectively, *p* = 0.013 (Table 3)). It is finally of note that higher DII is predominantly associated with significantly less intake in the entire spectrum of micronutrients and vitamins (Table 3).

**Table 3:** Intakes of nutrients consumed by the subjects that were divided according to median DII. The table lists the dietary intakes of the nutrients consumed by the subjects with DII below the median (DII < median, N = 331) versus the subjects with DII over median (DII > median, N = 332)

Daily intake of nutrients	Total sample (n = 663)	DII < median (n = 331)	DII > median (n = 332)	
		Median (25th -75th percentiles)	Median (25th -75th percentiles)	p
**Energy intake (Kcal/day)**	1711.96 (1469.94-2045.29)	1882.61 (1587.64-2168.17)	1592.03 (1344-1848.5)	< 0.001
**Energy from macronutrients**:				
**Energy from lipids (%En/day)**	35.11 ± 5.61	34.73 ± 5.53	35.49 ± 5.68	0.083
**Energy from saturated fat (%En/day)**	11.56 ± 2.50	11.08 ± 2.36	12.05 ± 2.55	< 0.001
**Energy from monounsaturated fat (%En/day)**	16.25 (14.3-18.25)	16.25 (14.31–18.33)	16.26 (14.22–17.93)	0.647
**Energy from polyunsaturated fat (%En/day)**	4.04 (3.47–4.77)	4.12 (3.55–4.9)	3.88 (3.39–4.6)	0.002
**Energy from omega-3 polyunsaturated fat (%En/day)**	0.6 (0.5–0.73)	0.64 (0.53–0.77)	0.57 (0.49–0.69)	< 0.001
**Energy from omega-6 polyunsaturated fat (%En/day)**	3.32 (2.81-4)	3.42 (2.88–4.12)	3.24 (2.73–3.88)	0.013
**Energy from proteins (%En/day)**	16.04 (14.67–17.62)	16.04 (14.65–17.59)	16.04 (14.73–17.66)	0.553
**Energy from carbohydrates (%En/day)**	50 ± 6.27	50 ± 6.27	49.48 ± 6.36	0.284
**Energy from soluble carbohydrates (%En/day)**	16.66 (14.07–19.52)	17.21 (14.76–20.08)	15.84 (13.29–18.96)	< 0.001
**Daily intake of micronutrients**:				
**Calcium (mg/day)**	638.21 (503.51-782.09)	713.53 (568.12-847.42)	569.42 (474.34-705.86)	< 0.001
**Iron (mg/day)**	9.74 (7.93–11.58)	11.09 (9.57–12.67)	8.11 (7.07–10.08)	< 0.001
**Sodium (mg/day)**	1772.92 (1411.09-2275.05)	1927.06 (1519.7-2425.92)	1671.76 (1308.76-2075.68)	< 0.001
**Potassium (mg/day)**	2596.59 (2192.95-2990.21)	2931.34 (2653.53-3266.4)	2223.86 (1985.62-2503.77)	< 0.001
**Phosphorus (mg/day)**	1053.82 (889.49-1234.9)	1154.56 (988.41-1305.85)	959.42 (834.21-1112.65)	< 0.001
**Zinc (mg/day)**	8.96 (7.71–10.53)	9.82 (8.57–11.4)	8.22 (7.13–9.34)	< 0.001
**Magnesium (mg/day)**	156.2 (129.01-186.14)	172.57 (145.16-203.69)	139.48 (116.32-164.95)	< 0.001
**Selenium (mg/day)**	29.87 (22.1-40.26)	33.7 (25.51–44.18)	26.04 (19.84–35.19)	< 0.001
**Vitamin B1 (mg/day)**	0.89 (0.75–1.08)	1.01 (0.86–1.15)	0.79 (0.68–0.94)	< 0.001
**Vitamin B2 (mg/day)**	1.4 (1.17–1.64)	1.58 (1.38–1.78)	1.25 (1.08–1.43)	< 0.001
**Vitamin C (mg/day)**	108.49 (75.8-151.17)	139.24 (109.92-177.62)	80.62 (59.38-107.27)	< 0.001
**Vitamin B3 (mg/day)**	16.57 (14.19–19.75)	18.36 (15.6-20.99)	15.35 (12.94–17.82)	< 0.001
**Vitamin B6 (mg/day)**	1.62 (1.37–1.89)	1.83 (1.61–2.04)	1.43 (1.24–1.64)	< 0.001
**Folates (**µ**g/day)**	256.19 (209.88-310.85)	302.4 (257.66-345.18)	219.93 ± 56.29	< 0.001
**Pantothenic acid (mg/day)**	2.48 (2.03–3.01)	2.79 (2.34–3.35)	2.23 (1.89–2.62)	< 0.001
**Biotin (mg/day)**	16.66 (13.54–20.43)	18.78 (15.05–22.51)	15.22 (12.51–17.86)	< 0.001
**Vitamin B12 (**µ**g/day)**	4 (2.96-6)	4.39 (3.22–6.85)	3.63 (2.78–5.05)	< 0.001
**Vitamin A (RE/day)**	718.38 (547.99–941.6)	862.68 (721.98-1095.18)	575.93 (476.17-713.22)	< 0.001
**Vitamin E (**µ**g/day)**	9.7 (8.07–11.77)	11.23 (9.44–13.07)	8.72 ± 2.38	< 0.001
**Vitamin D (**µ**g/day)**	2.18 (1.38–4.23)	3.01 (1.58–4.83)	1.75 (1.16–3.13)	< 0.001
**Vitamin K (**µ**g/day)**	6.78 (2.39–13.55)	8.46 (3.39–15.71)	5.61 (1.94–10.99)	< 0.001
**B-carotene (**µ**g/day)**	2779.36 (2032.86-4026.24)	3646.14 (2822.11-4735.32)	2109.04 (1592.32-2659.43)	< 0.001
**Alcohol (g/day)**	5.9 (0.85–17.81)	9.19 (1.7–20.5)	4.16 (0.34–14.41)	< 0.001
**Caffeine (g/day)**	0.1 (0.06–0.15)	0.11 (0.06–0.16)	0.1 (0.06–0.15)	0.085

### Higher Dietary Inflammatory Index is associated with plasma markers of inflammation

**Fig. 1:**
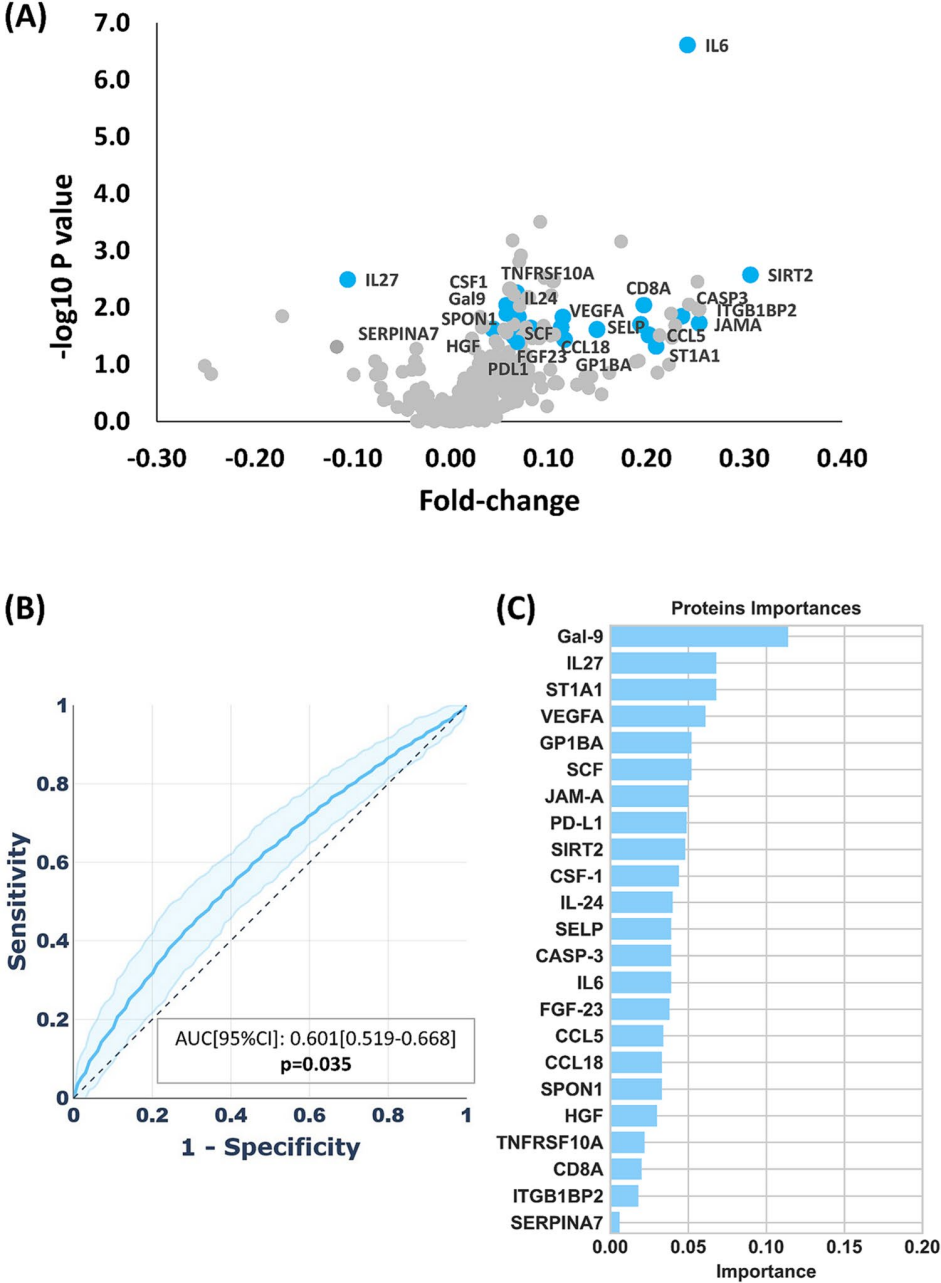
Higher DII associates with variations in the plasmatic expression of multiple inflammatory proteins. (**A**) Volcano plot, showing how much the plasmatic expression of each of the 368 proteins in subjects with DII > median changes versus the plasmatic expression of the same protein in subjects with DII < median. Data are expressed as fold of changes in log_2_ scale on the x axis and as–log10 p value on the y axis. (**B**) Receiving Operating Curve (ROC) reporting the performance of the machine learning model (as sensitivity and 1-specificity to detect subjects with DII > median including the 368 proteins measured in plasma. The Area Under the Curve (AUC), the upper and lower limits of the 95% confidence interval and the p-value are reported. (**C**) Random forest classifier plot showing, in descending order, the relative importance of the top predictors for DII > median by the machine learning model

Subjects with DII > median presented with higher CRP levels versus subjects with DII < median (0.10 (0.06–0.07) vs. 0.08 (0.04–0.15) mg/L respectively, *p* = 0.004; Table 1), and with higher plasmatic NPXs of 61 proteins but lower plasmatic NPXs of 3 proteins (Fig. 1A; Supplemental Table 1 reports the mean and the standard errors of each protein in both groups, the p values and the log2fold of change, which indicates how much the NPX of each protein changes, on average, in the subjects with DII > median compared to subjects with DII < median).

Next, to identify which of these proteins mostly contribute to variations in DII, we employed a machine learning boosting prediction model. This model, trained on a subset of 194 subjects with DII > median versus 203 subjects with DII < median (“training sets”), was then tested in an internal “test set” (138 subjects with DII > median vs 128 subjects with DII < median; see methods) to identify the most important contributors for the increase of DII values. This model, which achieved significant performance in discriminating subjects with DII > median versus subjects DII < median in the test set (Area Under the Curve (AUC) of Receiver Operating Characteristic (ROC) = 0.601 (0.519–0.668), and *p* = 0.035) (Fig. 1B), underscored 23 most representative proteins (listed in Fig. 1C in descending order of importance). Out of these proteins, 22 displayed increased plasmatic NPX in subjects with DII > median versus subjects with DII < median, and included Galectine-9 (Gal9), Sulfotransferase 1A1 (ST1A1), Vascular Endothelial growth factor A (VEGFA), Platelet glycoprotein Ib alpha chain (GP1A1), Stem cell factor (SCF), Junctional adhesion molecule A (JAM-A), Programmed death-ligand 1 (PDL1), Sirtuin-2 (SIRT2), Colony Stimulating Factor 1 (CSF-1), Interleukin-24 (IL-24), Interleukin-6 (IL-6), Selectin-P (SELP), Caspase 3 (CASP3), Fibroblast Growth Factor 3 (FGF-23), Chemokine-ligand 5 (CCL5), Chemokine-ligand 18 (CCL18), Spondin-1 (SPON1), Hepatocyte Growth Factor (HGF), Tumor Necrosis Factor Receptor Superfamily member 10A (TNFRSF10A), CD8 subunit alpha (CD8A), Integrin Subunit Beta 1 Binding Protein 2 (ITGB1BP2), Serpin Family A Member 7 (SERPINA7). By contrast, only Interleukin-27 (IL-27) was significantly reduced in subjects with DII > median vs subjects with DII < median.

Finally, by Gene Ontology enrichment analysis we found that these 23 proteins are significantly clustered into up to 52 biological processes (“GO_bp”). Of them, 24 are related to immune-inflammatory pathways (red bars in Supplemental Fig. 2), 23 refer to cell-cell signaling pathways (grey bars in Supplemental Fig. 2) and 5 are involved in metabolic processes (blue bars in Supplemental Fig. 2). A detailed list of these biological processes, with their folds of enrichments and FDR, is available as Supplemental Table 3.

## Discussion

Our findings contribute to a better understanding of the inflammatory consequences of unhealthy dietary habits, which are a risk factor for the development of obesity, cardiometabolic, and cardiovascular diseases. In fact, higher DII did not only associate with increased levels of a clinically used marker of low-grade inflammation, the CRP (a finding that is in line with some data from literature [13–46, 47] but in contrast with others [48]), but it also reflected significant variations in the plasmatic abundance of multiple inflammatory proteins, out of one of the largest arrays measured in this field and that we previously associated with increased cardiovascular risk [27, 28].

Indeed, two previous studies, which measured a smaller number of biomarkers with the same PEA technology, found associations between several proteins with either unhealthy dietary patterns (21/184 proteins in one study [6]) or with increased DII (55/163 proteins in another one [30]). By contrast, in our study of the NPXs up to 61 proteins were increased and 3 were reduced in subjects with higher DII versus subjects with lower DII. Our machine training learning model restricted the importance to 23 of them, 22 of which, including pro-inflammatory proteins, presented with increased plasmatic NPXs, while only IL-27, a protein known of immunoregulatory potential [49], was reduced in subjects with higher DII. 6 proteins that were found associated with DII in the second study (VEGFA, PDL1, IL6, FGF23, HGF and CD8A) were also detected in our study. In addition, we have identified a number of other proteins associated with metabolic pathways which are consistent with a pro-inflammatory effect of diet with high DII. The fact that none of these pathways was previously identified may depend upon the different panels tested in the different studies and the different methodologies used. Therefore, our study adds new information to what previously reported by others and expands the reach of dietary effects on the overall biological pathways related to inflammation. Anyhow, we cannot rule out that increasing the number of biomarkers might allow to find even further pathways. Indeed, two other studies, which measured a larger number of proteins compared to our work using an alternative technology (4,955 in one study [4] and 1,713 in another [5]), found a significant association between dietary patterns, evaluated by qualitative food frequency questionnaires, with 20 and 5 proteins respectively.

Higher DII was associated with the intake of only some macronutrients, while it was predominantly reflected a lower intake in the entire spectrum of micronutrients and vitamins which, although not providing energetic supply, significantly contribute to the “inflammatory effect score” used to estimate their anti-inflammatory potential [18]. We thereby speculate that a plausible inflammatory effect of diet should be investigated considering the broader concept of the “food matrix” [50], as a sum of multiple nutritional components of a food consumed, rather than focusing on the intake of some macronutrients, for instance, dietary fats, whose relationship with the odds of developing cardiometabolic and cardiovascular diseases is still currently debated [51, 52]. This possibility can be achieved only through the analysis of the quantitative seven-days dietary records, but not with the qualitative FFQs, commonly used in large epidemiological studies [8, 13–16]. Indeed, these tools are affected by significant shortcomings, like lacking standardizations and limited accuracy of the dietary assessments relying on publicly available biobanks (including the ones for the Italian population [53]) and used to calculate scores/indices of healthy/unhealthy dietary patterns (e.g., the PREDIMED score [54]). Although we acknowledge that the seven days dietary records could be representative of the adherence to a specific dietary pattern in a limited timeframe, we are confident about the quality of the dietary information gathered with using this methodological approach, as testified by the total caloric intakes, which were in line with the current dietary surveys for the Italian population [53]. Anyhow, multiple aspects related to diet (e.g. the geographic locations [55], the socioeconomic status [56], the processing and quality of foods [50]) could significantly impact and cannot be unmasked in this single-center experience. Validation studies in independent cohorts and in subjects with more advanced cardio-metabolic impairment are warranted.

We also acknowledge other limits in our study. First, the PEA technology, employed for this proteomics analysis, although ensuring an elevated degree of sensitivity, provides information of a relative abundance (NPX values [29]), but not of an absolute quantity. Therefore, the future step of our study will be to confirm such data of abundance into absolute quantities by techniques of mass-spectrometry.

Finally, longitudinal studies still demonstrated that dietary changes towards adherence to healthier dietary patterns result into reductions of DII [57, 58], and whether such changes also lead to reductions in the plasma abundance of inflammatory proteins will be a matter of future analyses.

## Conclusions

Higher DII, calculated from the quantitative analysis of the consumption of specific food patterns and nutritional intakes, associates with significant variation of a large set of inflammatory proteins in plasma.

### Electronic supplementary material

Below is the link to the electronic supplementary material.

Supplementary Material 1

Supplementary Material 2

Supplementary Material 3

## Data Availability

The pooled data that support the findings of this study are available from the author A.L.C., upon reasonable request.

## Abbreviations

DII: Dietary Inflammatory Index
CRP: C-Reactive Protein
Gal-9: Galectine-9
ST1A1: Sulfotransferase 1A1
VEGFA: Vascular Endothelial growth factor A
GP1A1: Platelet glycoprotein Ib alpha chain
SCF: Stem cell factor
JAM-A: Junctional adhesion molecule A
PDL1: Programmed death-ligand 1
SIRT2: Sirtuin-2
CSF1: Colony Stimulating Factor 1
IL-24: Interleukine-24
IL-6: Interleukine-6
SELP: Selectin-P
CASP3: Caspase 3
FGF23: Fibroblast growth factor 3
CCL5: Chemokine-ligand 5
CCL18: Chemokine-ligand 18
SPON1: Spondin-1
HGF: Hepatocyte growth factor
TNFRSF10A: tumor necrosis factor receptor superfamily member 10 A
CD8A: CD8 subunit alpha
ITGB1BP2: Integrin Subunit Beta 1 Binding Protein 2
SERPINA7: Serpin Family A Member 7
IL-27: Interleukin-27
