# Supplementary material for: High dietary inflammatory index associates with inflammatory proteins in plasma
Source: Diabetol Metab Syndr. 2024 Feb 26;16:50. doi: 10.1186/s13098-024-01287-y (PMC10895728; doi:10.1186/s13098-024-01287-y)
Supplement: Supplementary file 1 — Supplementary Material 1 [file 13098_2024_1287_MOESM1_ESM.docx]

**Supplemental Material**

**Methods and statistics**

**Ethic statements**

The PLIC study, including clinical assessments, collection of biological samples and analysis of individual data, was approved in 2001 by the Scientific Ethic Committee of the University of Milan (SEFAP/Pr.0003). Informed consent was obtained from subjects (all over 18 years-old), in accordance with the Declaration of Helsinki. Data management and statistical analyses were performed with the coordination of the Epidemiology and Preventive Pharmacology Centre (SEFAP) of the University of Milan.

**Selection criteria**

We considered the following exclusion criteria at baseline visit: (i) documented or reported CVD (either ischemic heart disease, ST elevation or non-ST elevation myocardial infarction, aortic-coronary by-pass grafting, angioplasty, transient ischemic attack, stroke, heart failure from Class II to IV according to New York Heart Association (NYHA), definition or documented peripheral arteriopathy), (ii) presence of pre-clinical atherosclerosis documented by imaging in carotid or other vascular territories (iii) documented Chronic Kidney Disease (Glomerular Filtration Rate, GFR < 60 ml/min, documented albuminuria > 30 mg/day) or any other renal diseases (iv) reported malignancies and (v) being pregnant. 1806 subjects were free from these criteria.

**Assessment of selection bias**

Assessment of selection bias was performed by Kolmogorov-Smirnov test, comparing the mean distribution of the linear variables in the selected study group (n=663) versus the original PLIC cohort. Chi-square test was performed to compare the dichotomous variables. The 663 subjects included in the analysis were representative of the entire group of the PLIC study for the biochemical characteristics included in this study. Anyhow, they were not comparable regarding: i) age, as the subjects of the selected sample were older as compared to the entire PLIC cohort (54.67±8.09 vs 48.71±11.4 years, *p*<0.001); ii) gender, as there was a higher prevalence of women in the selected cohort as compared to the original PLIC cohort (65.19 % vs 57.57%, *p*=0.001); iii) waist/hip ratio, which was lower in the selected cohort (0.82 (0.79-0.86) vs 0.86 (0.81-0.93), *p*<0.001)); iv) fasting glucose, which was significantly higher among subjects of the entire PLIC cohort vs. those of the study sample (90 (83-98) vs 88 (82-95) mg/dL, *p*=0.001). Finally, prevalence of active physical activity was higher in the study sample (45.36% vs 38.02%, *p*=0.003).

**Proteomics analysis**

Proteins were measured in 200 uL of plasma samples by Proximity Extension Assay (PEA). During the basal visit, plasma samples were separated from whole blood after centrifugation and immediately frozen at -80 degrees in our core lab, as previously described ^1^. Then, after the end of the follow-up of this study, aliquots were thawed and plasma was transferred, on ice, to 96-well plates and shipped to Olink^TM^ proteomics AB (Uppsala, Sweden) on dry ice for proteomic analysis. Manufacturer’s instruction and assistance during this preparation guarantee that a single thawing cycle from the original collection does not affect the quality of the sample and the standard deviation (SD) of the analysis. The final assay read-out is given in Normalized Protein eXpression (NPX), which is an arbitrary unit on log2-scale where a high value corresponds to a higher protein expression. Each PEA measurement has a lower detection limit (LOD) calculated based on negative controls that are included in each run, and measurements below LOD were removed from further analysis. Using an internal extension control and an interpolate control, data quality is controlled and normalized. All assay characteristics including detection limits and measurements of assay performance and validations are available from the manufacturer’s webpage (http://www.olink.com).

**Sample size statistical analysis**

To support the statistical power of our analyses, this sample size was verified estimating according to similar previous experiences ^2^, investigating the relation between the inflammatory potential of diet and CRP plasma levels. Therefore, setting alpha=0.05 and power=0.80, 612 replicates are at least required to support robust statistical power (F-test, ANOVA fixed effects, omnibus, one-way, G*power 3.1.9.7 (http://gpower.hhu.de/)).

**Supplemental references**

1. Hoogeveen RM, Pereira JPB, Nurmohamed NS, Zampoleri V, Bom MJ, Baragetti A, Boekholdt SM, Knaapen P, Khaw K-T, Wareham NJ, et al. Improved cardiovascular risk prediction using targeted plasma proteomics in primary prevention. *Eur Heart J* [Internet]. 2020;Available from: https://pubmed.ncbi.nlm.nih.gov/32808014/

2. Suzuki K, Shivappa N, Kawado M, Yamada H, Hashimoto S, Wakai K, Iso H, Okada E, Fujii R, Hébert JR, et al. Association between dietary inflammatory index and serum C-reactive protein concentrations in the Japan Collaborative Cohort Study. *Nagoya J Med Sci* [Internet]. 2020 [cited 2023 Aug 31];82:237–249. Available from: https://pubmed.ncbi.nlm.nih.gov/32581404/

**Supplemental Figure legends.**

**Supplemental Figure 1- Study Design, train, and test of the machine learning model.**

**Supplemental Figure 2- Biological processes represented by the 23 most important proteins associated with higher DII.**

Bar plot reporting on the y-axis the biological processes from gene ontology analysis clustered by their major biological involvement (red: inflammation, grey: cellular processes of molecular signaling, blue: metabolism). On the x-axis is reported the statistical significance as negative logarithm (base 10) of the False Discovery Rate (FDR).

**Supplemental Table legends.**

**Supplemental Table 1 - Intakes of food groups and subgroups reported to be consumed by the subjects divided according to median DII.**

The table lists the quantities of food groups (in bold) and subgroups consumed by the subjects with DII below the median (DII<median, N=331) versus the subjects with DII over median (DII>median, N=332). “NC” indicates a food subgroup that was checked in the dataset but it was not consumed by any participant.

**Supplemental Table 2 - Comparison of the proteins NPX and their variations, as fold change, between subjects in the subjects divided according to median DII.**

The table reports the absolute NPX values of each protein measured with the targeted proteomics panels (see methods for details) in the plasma of subjects with DII below versus subjects with DII over the median value. For each protein, the mean value and the standard deviation is reported. The Fold of change is calculated in order to measure the variation of each protein in the plasma of subjects with DII over the median as compared to the mean value of that protein in the subjects with DII below the median. The last column of the right indicates “Increased” is the fold of change is positive (protein increased in the plasma of subjects with DII over the median) or “Decreased” is the fold of change is negative (protein increased in the plasma of subjects with DII over the median). The p value is derived from the statistical comparison (t-test) of the mean NPX values of both groups.

**Supplemental Table 3- Biological processes identified by gene ontology enrichment analysis to be represented by the 23 proteins that were identified by the machine learning model.**

The table reports the biological processes (“GO_bp”) predicted by gene ontology analysis including the 23 proteins that were identified by the machine learning model. For each GO_bp the fold of enrichment, the p-value, the Fals Discovery Rate (FDR) and the involved inflammatory proteins are reported.

**Supplemental Table 1- Intakes of food groups and subgroups reported to be consumed by the subjects divided according to median DII.**

|  | **Total sample (n=663)** | **DII<median (n=331)** | **DII>median (n=332)** |  |
| --- | --- | --- | --- | --- |
|  | *Median (25th-75th percentiles)* | *Median (25th-75th percentiles)* | *Median (25th-75th percentiles)* | *p* |
| **Tubers, potatoes, starch (g/day)** | **21.43 (6.89-40)** | **24.07 (7.14-44.84)** | **19.01 (5.63-35.35)** | **0.019** |
| **Vegetables, mushrooms (g/day)** | **200.21 (147.14-254.69)** | **234.57 (191.04-295.65)** | **164.14 (117.87-209.18)** | **<0.001** |
| Sprouts/shoots (soya, bamboo), asparagus (g/day) | 0 (0-0) | 0 (0-0) | 0 (0-0) | 0.064 |
| Root vegetables (g/day) | 7.6 (2.13-21.87) | 13.57 (4.17-29.29) | 4.48 (0.82-12.14) | <0.001 |
| Cruciferous vegetables (g/day) | 0 (0-14.29) | 4.29 (0-22.5) | 0 (0-8.68) | <0.001 |
| Allium vegetables (g/day) | 7.14 (2.86-14.29) | 8.78 (4.29-17.29) | 5.71 (2.5-11.4) | <0.001 |
| Vegetable juices (g/day) | 0 (0-0) | 0 (0-0) | 0 (0-0) | 0.313 |
| Mushrooms, truffles (g/day) | 0 (0-1.07) | 0 (0-2.86) | 0 (0-0.36) | 0.552 |
| Leafy vegetables (including salads) (g/day) | 46.16 (25.69-71.43) | 57.14 (34.29-82.44) | 37.14 (19.47-57.14) | <0.001 |
| Fruit and flower vegetables (celery, fennels, artichokes, peppers, etc) (g/day) | 97.31 (64.34-145.71) | 120 (82-157.86) | 82.86 (54.76-120.36) | <0.001 |
| Olives, vegetables preserved in oil or vinegar (g/day) | 0 (0-2.86) | 0 (0-2.86) | 0 (0-2.86) | 0.860 |
| Seaweeds (g/day) | NC | NC | NC | - |
| **Legumes and soy products (g/day)** | **12.86 (0-28.57)** | **14.29 (3.17-33.33)** | **5.7 (0-21.43)** | **<0.001** |
| Mixed vegetables and legumes (g/day) | 0 (0-0) | 0 (0-0) | 0 (0-0) | 0.129 |
| Legumes (g/day) | 11.59 (0-28.57) | 14.29 (2.86-31.75) | 5.24 (0-21.43) | <0.001 |
| Legume flours (g/day) | 0 (0-0) | 0 (0-0) | 0 (0-0) | 0.317 |
| Soybean products (g/day) | 0 (0-0) | 0 (0-0) | 0 (0-0) | 0.090 |
| **Fresh fruits, dried fruits, flours, juices (g/day)** | **261.07 (168.78-350.07)** | **307.14 (238.57-411.43)** | **214.29 (119.86-301.35)** | **<0.001** |
| Fresh fruit and berries (g/day) | 238.21 (142.14-323.06) | 285.71 (207.14-370) | 190.99 (89.29-278.57) | <0.001 |
| Cooked or canned fruit (g/day) | 0 (0-0) | 0 (0-0) | 0 (0-0) | 0.157 |
| Dried fruit and seeds (g/day) | 0 (0-2.86) | 0.71 (0-4.29) | 0 (0-1.79) | <0.001 |
| Fruit flours (chestnuts, coconut, etc) (g/day) | 0 (0-0) | 0 (0-0) | 0 (0-0) | 0.318 |
| Fruit juices and drinks (mL/day) | 0 (0-28.57) | 0.71 (0-31.25) | 0 (0-4.23) | <0.001 |
| **Milk and yogurt (mL/day)** | **172.86 (99.34-235.36)** | **182.14 (110-249.29)** | **165.36 (92.14-222.6)** | **0.016** |
| Milk (mL/day) | 122.85 (28.12-170) | 125 (27.86-187.86) | 110.41 (27.61-162.41) | 0.061 |
| Yogurt (mL/day) | 0 (0-35.71) | 0 (0-42.86) | 0 (0-21.21) | 0.059 |
| Milk flour, condensed or powdered milk (g/day) | 0 (0-0) | 0 (0-0) | 0 (0-0) | 0.997 |
| **Cheeses (g/day)** | **34.15 (21.43-47.47)** | **33.85 (20.71-47.81)** | **34.27 (23.03-47.5)** | **0.348** |
| **Cereals, flours, pasta, bread, crackers, rusks (g/day)** | **162.33 (122-207.5)** | **174.8 (131.67-218.57)** | **154.29 (113.1-197.57)** | **<0.001** |
| Cereals in grain and products (g/day) | 12 (5.71-22.86) | 12.86 (7.14-22.86) | 11.43 (1.43-22.86) | 0.150 |
| Cereal flour and starch (g/day) | 1.44 (0-11.43) | 0.82 (0-9.51) | 2.56 (0-12.24) | 0.080 |
| Cereal flakes, popcorns, bran (also in tablets), popped (g/day) | 0 (0-0) | 0 (0-0) | 0 (0-0) | <0.001 |
| Pasta (g/day) | 42.9 (28.25-61.94) | 42.86 (30-62.86) | 44.29 (27.14-61.43) | 0.470 |
| Filled pasta ("ravioli" and "tortellini") (g/day) | 0 (0-0) | 0 (0-0) | 0 (0-0) | 0.128 |
| "Grissini", crackers, salted snacks, "crostini" (g/day) | 0 (0-2.14) | 0 (0-4.29) | 0 (0-0) | <0.001 |
| Bread, toasted bread, pizza, focaccia, bread, and pizza dough (g/day) | 85.71 (51.9-114.74) | 92.86 (60.5-122.14) | 72.86 (46.34-105.71) | <0.001 |
| **Processed meats (g/day)** | **24.43 (13.53-37.4)** | **25.71 (13.57-37.23)** | **23.09 (13.42-37.71)** | **0.460** |
| **Meat and offal (g/day)** | **67.78 (44.29-91.34)** | **68.57 (48.95-93.01)** | **64.29 (42.86-89.29)** | **0.112** |
| Equine meat (horse and donkey) (g/day) | 0 (0-0) | 0 (0-0) | 0 (0-0) | 0.495 |
| Other meats (not bovine, ovine, suine, equine, birds, rabbits) (g/day) | 0 (0-0) | 0 (0-0) | 0 (0-0) | 0.090 |
| Equine offals (g/day) | NC | NC | NC |  |
| Bovine meat (adult animal) (g/day) | 27.35 (14.19-45.13) | 28.57 (14.29-48.57) | 25.57 (13.46-44.11) | 0.626 |
| Veal meat (g/day) | 0 (0-0) | 0 (0-0) | 0 (0-0) | 0.372 |
| Canned meats (g/day) | 0 (0-0) | 0 (0-0) | 0 (0-0) | 0.643 |
| Bovine offals (g/day) | 0 (0-0) | 0 (0-0) | 0 (0-0) | 0.964 |
| Poultry, birds (g/day) | 17.14 (0-36.28) | 18.57 (10-35.71) | 15.75 (0-37.32) | 0.158 |
| Poultry offals, paté (g/day) | 0 (0-0) | 0 (0-0) | 0 (0-0) | 0.025 |
| Pork (suine) meat (g/day) | 0 (0-1.1) | 0 (0-0.85) | 0 (0-1.37) | 0.839 |
| Pork (suine) offals (g/day) | 0 (0-0) | 0 (0-0) | 0 (0-0) | 0.317 |
| Rabbit, hare meat (g/day) | 0 (0-0) | 0 (0-0) | 0 (0-0) | 0.879 |
| Rabbit, hare offals (g/day) | 0 (0-0) | 0 (0-0) | 0 (0-0) | 0.318 |
| Ovine meat (sheep, lamb, goat) (g/day) | 0 (0-0) | 0 (0-0) | 0 (0-0) | 0.664 |
| Ovine offals (g/day) | 0 (0-0) | 0 (0-0) | 0 (0-0) | 0.253 |
| **Fish, crustaceans, mollusks (g/day)** | **28.57 (14.29-50)** | **35.71 (20.71-60)** | **25.36 (7.14-41.79)** | **<0.001** |
| Finfish (fresh, frozen or cooked) (g/day) | 17.14 (0-31.51) | 21.43 (7.14-38.57) | 14.29 (0-28.57) | <0.001 |
| Mollusks (fresh, frozen, cooked, canned or preserved) (g/day) | 0 (0-7.14) | 0 (0-11.43) | 0 (0-3.16) | 0.001 |
| Finfish (canned in oil) (g/day) | 0 (0-5.76) | 0 (0-7.14) | 0 (0-2.94) | 0.048 |
| Finfish (canned or preserved) and fish egg (g/day) | 0 (0-0) | 0 (0-0) | 0 (0-0) | 0.169 |
| Crustaceans (fresh, frozen, cooked, canned or preserved) (g/day) | 0 (0-0) | 0 (0-0) | 0 (0-0) | 0.137 |
| **Eggs (g/day)** | **8.33 (1.75-14.37)** | **8.57 (2.14-16.43)** | **7.41 (1.53-14.29)** | **0.240** |
| **Oils, margarines, butter, cream (g/day)** | **27.86 (20.69-35.71)** | **30 (23.04-38.35)** | **26.24 (18.81-32.63)** | **<0.001** |
| Margarine (g/day) | 0 (0-0) | 0 (0-0) | 0 (0-0) | 0.771 |
| Oils and vegetable fats (including peanut butter and vegetable margarine) (mL/day) | 22.86 (17.14-29.14) | 25.17 (19.06-32.33) | 20.71 (14.58-25.96) | <0.001 |
| Butter and animal fats (excluding cream) (mL/day) | 2.86 (0-6.43) | 2.86 (0-6.43) | 2.85 (0-6.31) | 0.888 |
| Cream (mL/day) | 0 (0-0) | 0 (0-0) | 0 (0-0) | 0.950 |
| **Sweets, sugar, jams, ice creams (g/day)** | **20.32 (9.66-37.39)** | **21.43 (10.31-41.43)** | **19.29 (9.29-34.86)** | **0.186** |
| Nougat (g/day) | 0 (0-0) | 0 (0-0) | 0 (0-0) | 0.968 |
| Chocolate candies, chocolate bars and spreads (g/day) | 0 (0-2.86) | 0 (0-2.86) | 0 (0-2.86) | 0.884 |
| Candied fruits (g/day) | 0 (0-0) | 0 (0-0) | 0 (0-0) | 0.257 |
| Sugar and honey (g/day) | 8.79 (3.38-16.04) | 8.71 (3.17-15.86) | 8.71 (3.65-16.43) | 0.907 |
| Candies, licorice, sugar-coated almonds (g/day) | 0 (0-0) | 0 (0-0.14) | 0 (0-0) | 0.022 |
| Jams, marmalade (g/day) | 0 (0-0) | 0 (0-1.43) | 0 (0-0) | 0.067 |
| Ice creams, ice pops (g/day) | 0 (0-17.32) | 0 (0-21.43) | 0 (0-13.81) | 0.045 |
| Artificial sweeteners (g/day) | 0 (0-0) | 0 (0-0) | 0 (0-0) | 0.032 |
| Syrups (mL/day) | 0 (0-0) | 0 (0-0) | 0 (0-0) | . |
| **Croissants, cookies, puddings, cakes (g/day)** | **35.71 (20-59.55)** | **39.29 (20.29-61.43)** | **33.47 (19.88-55.71)** | **0.088** |
| Buns, rolls (g/day) | 0 (0-8.57) | 0 (0-8.57) | 0 (0-8.57) | 0.196 |
| Melba toasts (g/day) | 0 (0-8.29) | 0 (0-9.29) | 0 (0-6.86) | 0.058 |
| Puddings, spoon-desserts (mL/day) | 0 (0-0) | 0 (0-0) | 0 (0-0) | 0.759 |
| Pastries (excluding dry pastries) (g/day) | 0 (0-0) | 0 (0-0) | 0 (0-0) | 0.266 |
| Cakes and cake mixes (g/day) | 0 (0-15) | 4.29 (0-18.57) | 0 (0-12.26) | 0.002 |
| Cookies, biscuits and dry pastries (g/day) | 8.27 (0-24.14) | 8.57 (0-25) | 7.77 (0-22.5) | 0.662 |
| Buns, rolls, doughnuts (also filled or coated) (g/day) | 0 (0-0) | 0 (0-0) | 0 (0-0) | 0.919 |
| Chocolate-based snacks (like mars, kit-kat) (g/day) | NC | NC | NC | - |
| **Non-alcoholic and alcoholic beverages, cocoa, coffee, tea, infusions (mL/day)** | **247.94 (132.03-379.17)** | **275 (149.29-388.57)** | **211.92 (117.66-356.71)** | **0.002** |
| Non-alcoholic beverages (mL/day) | 0 (0-17.86) | 0 (0-10.5) | 0 (0-25.54) | 0.492 |
| Wine and sparkling wines (mL/day) | 47.2 (4.29-160.71) | 85.71 (8.57-196.43) | 32.73 (2.06-113.39) | <0.001 |
| Sweet and bitter liquors (mL/day) | 0 (0-0) | 0 (0-0) | 0 (0-0) | 0.301 |
| Beer (mL/day) | 0 (0-24.29) | 0 (0-21.43) | 0 (0-28.57) | 0.953 |
| Distillates, high-alcohol beverages, and high-alcohol bitter liquors (mL/day) | 0 (0-0) | 0 (0-0) | 0 (0-0) | 0.705 |
| Ethanol (mL/day) | NC | NC | NC | - |
| Cider (mL/day) | NC | NC | NC | - |
| Tea (also decaffeinated and instant) (mL/day) | 0 (0-64.29) | 0 (0-64.29) | 0 (0-64.29) | 0.503 |
| Coffee (also decaffeinated and instant) (mL/day) | 63.5 (38.57-90) | 64.29 (38.57-90) | 61.9 (38.57-85.71) | 0.375 |
| Cocoa and other powders (g/day) | 0 (0-0) | 0 (0-0) | 0 (0-0) | 0.727 |
| Herbal teas, infusions (also instant) (mL/day) | 0 (0-0) | 0 (0-0) | 0 (0-0) | 0.396 |
| Barley coffee, chicory coffee (also ready to drink) (mL/day) | 0 (0-0) | 0 (0-0) | 0 (0-0) | 0.107 |
| **Aromatic herbs, spices (g/day)** | **15.2 (4.05-36.53)** | **15.29 (4.64-35.71)** | **15.09 (3.01-37.14)** | **0.410** |
| **Miscellaneous (g/day)** | **14.00 (3.00-36.00)** | **14.00 (3.00-36.00)** | **14.00 (2.00-36.00)** | **0.585** |
| Vinegar, grape or apple (mL/day) | 0 (0-2.86) | 0 (0-2.86) | 0 (0-2.14) | 0.090 |
| Soup cubes, extracts, Monosodium glutamate products (g/day) | 0 (0-0.25) | 0 (0-0.29) | 0 (0-0.13) | 0.306 |
| Sauces, based on mayonnaise (mL/day) | 0 (0-0) | 0 (0-0) | 0 (0-0) | 0.422 |
| Raising agents (yeast, baking powder) (g/day) | 0 (0-0) | 0 (0-0) | 0 (0-0) | 0.792 |
| Broth, meat and vegetable (g/day) | 7.6 (0-33.54) | 7.71 (0-31.43) | 6.85 (0-35.71) | 0.783 |
| Sauces (ketchup, "pesto", tuna sauce, walnut sauce) (g/day) | 0 (0-0) | 0 (0-0) | 0 (0-0) | 0.318 |
| Meal substitutes (g/day) | NC | NC | NC | - |
| Salt (g/day) | 0 (0-0) | 0 (0-0) | 0 (0-0) | 0.913 |
| "Sofficini" (breaded and fried stuffed crepes, commercial) (g/day) | 0 (0-0) | 0 (0-0) | 0 (0-0) | 0.179 |

**Supplemental Table 2- Comparison of the proteins NPX and their variations, as fold change, between subjects in the subjects divided according to median DII.**

| **Proteins** | **Mean NPX (DII<median)** | **S.D. NPX (DII<median)** | **Mean NPX (DII>median)** | **S.D. NPX (More DII>median)** | **Fold change** | **p** | **increased/decreased** |
| --- | --- | --- | --- | --- | --- | --- | --- |
| **IL6** | 2.508 | 0.645 | 2.750 | 0.692 | 1.183 | <0.001 | Increased |
| **CD5** | 4.144 | 0.385 | 4.235 | 0.363 | 1.066 | <0.001 | Increased |
| **IL17A** | 1.018 | 0.554 | 1.081 | 0.453 | 1.045 | 0.001 | Increased |
| **FABP4** | 5.453 | 0.648 | 5.627 | 0.663 | 1.128 | 0.001 | Increased |
| **RARRES2** | 11.038 | 0.292 | 11.110 | 0.294 | 1.051 | 0.001 | Increased |
| **LIF** | 0.767 | 0.466 | 0.837 | 0.426 | 1.050 | 0.002 | Increased |
| **SIRT2** | 3.650 | 1.106 | 3.956 | 1.281 | 1.236 | 0.003 | Increased |
| **PRSS8** | 8.546 | 0.417 | 8.642 | 0.411 | 1.069 | 0.003 | Increased |
| **IL27** | 4.131 | 0.449 | 4.026 | 0.464 | 0.930 | 0.003 | Decreased |
| **TRAILR2** | 4.455 | 0.326 | 4.560 | 0.542 | 1.075 | 0.003 | Increased |
| **LEP** | 5.800 | 1.201 | 6.052 | 1.098 | 1.191 | 0.003 | Increased |
| **IL10RB** | 5.936 | 0.313 | 5.997 | 0.309 | 1.043 | 0.005 | Increased |
| **TNFRSF10A** | 2.339 | 0.326 | 2.407 | 0.315 | 1.048 | 0.005 | Increased |
| **CD46** | 2.273 | 0.562 | 2.338 | 0.530 | 1.046 | 0.006 | Increased |
| **MCP3** | 1.684 | 0.453 | 1.787 | 0.493 | 1.074 | 0.006 | Increased |
| **PCSK9** | 3.145 | 0.390 | 3.225 | 0.360 | 1.057 | 0.007 | Increased |
| **FGF21_A** | 4.009 | 1.195 | 4.253 | 1.189 | 1.184 | 0.009 | Increased |
| **CSF1** | 8.682 | 0.264 | 8.739 | 0.276 | 1.040 | 0.009 | Increased |
| **CD8A** | 8.243 | 1.039 | 8.441 | 1.016 | 1.147 | 0.009 | Increased |
| **IL10** | 2.159 | 0.657 | 2.230 | 0.553 | 1.050 | 0.009 | Increased |
| **AXIN1** | 1.868 | 0.964 | 2.121 | 1.167 | 1.192 | 0.011 | Increased |
| **STAMBP** | 3.975 | 0.890 | 4.200 | 1.090 | 1.169 | 0.013 | Increased |
| **FGF23_A** | 1.784 | 0.527 | 1.841 | 0.511 | 1.040 | 0.013 | Increased |
| **CASP3** | 6.517 | 1.094 | 6.753 | 1.244 | 1.178 | 0.014 | Increased |
| **IGFBP1** | 5.593 | 0.995 | 5.421 | 1.082 | 0.888 | 0.014 | Decreased |
| **Gal9** | 8.098 | 0.355 | 8.161 | 0.324 | 1.045 | 0.014 | Increased |
| **IL24** | 1.264 | 0.647 | 1.334 | 0.589 | 1.049 | 0.015 | Increased |
| **IL17C** | 1.130 | 0.497 | 1.160 | 0.374 | 1.021 | 0.015 | Increased |
| **VEGFA** | 9.004 | 0.543 | 9.118 | 0.594 | 1.083 | 0.015 | Increased |
| **ITGB1BP2** | 3.089 | 0.967 | 3.342 | 1.189 | 1.192 | 0.019 | Increased |
| **CTSZ** | 5.182 | 0.376 | 5.246 | 0.400 | 1.046 | 0.020 | Increased |
| **JAMA** | 4.941 | 0.867 | 5.135 | 1.020 | 1.144 | 0.020 | Increased |
| **FGF21** | 5.018 | 1.265 | 5.248 | 1.278 | 1.172 | 0.021 | Increased |
| **CD59** | 1.890 | 0.334 | 1.924 | 0.294 | 1.024 | 0.021 | Increased |
| **SCF_A** | 9.951 | 0.484 | 10.046 | 0.421 | 1.068 | 0.021 | Increased |
| **IFNgamma** | 0.344 | 0.326 | 0.376 | 0.334 | 1.022 | 0.022 | Increased |
| **CCL18** | 5.994 | 0.691 | 6.107 | 0.676 | 1.081 | 0.022 | Increased |
| **TNFSF13B** | 6.868 | 0.340 | 6.945 | 0.341 | 1.055 | 0.022 | Increased |
| **SERPINA7** | 3.962 | 0.355 | 4.034 | 0.404 | 1.051 | 0.023 | Increased |
| **SCF** | 10.064 | 0.473 | 10.146 | 0.415 | 1.058 | 0.023 | Increased |
| **SPON1** | 0.767 | 0.235 | 0.810 | 0.258 | 1.031 | 0.024 | Increased |
| **CSTB** | 4.683 | 0.525 | 4.779 | 0.573 | 1.069 | 0.024 | Increased |
| **SELP** | 9.510 | 0.695 | 9.660 | 0.821 | 1.109 | 0.024 | Increased |
| **SOD2** | 8.533 | 0.301 | 8.588 | 0.321 | 1.039 | 0.024 | Increased |
| **HGF** | 8.275 | 0.465 | 8.348 | 0.463 | 1.052 | 0.027 | Increased |
| **TNFRSF9** | 5.960 | 0.401 | 6.017 | 0.399 | 1.040 | 0.028 | Increased |
| **FGF23** | 1.641 | 0.495 | 1.704 | 0.498 | 1.045 | 0.029 | Increased |
| **MMP12** | 5.252 | 0.603 | 5.358 | 0.646 | 1.076 | 0.030 | Increased |
| **CCL5** | 2.826 | 0.941 | 3.029 | 1.019 | 1.151 | 0.030 | Increased |
| **STK4** | 3.597 | 1.175 | 3.810 | 1.231 | 1.159 | 0.031 | Increased |
| **CD40** | 10.364 | 0.499 | 10.466 | 0.602 | 1.073 | 0.031 | Increased |
| **PDL1** | 4.642 | 0.479 | 4.707 | 0.419 | 1.046 | 0.032 | Increased |
| **CCL3_A** | 4.968 | 0.623 | 5.041 | 0.626 | 1.052 | 0.033 | Increased |
| **CD244** | 6.541 | 0.484 | 6.632 | 0.519 | 1.065 | 0.033 | Increased |
| **SCGB3A2** | 2.727 | 0.766 | 2.953 | 1.080 | 1.170 | 0.034 | Increased |
| **HSP27** | 9.544 | 0.592 | 9.628 | 0.556 | 1.060 | 0.034 | Increased |
| **ARTN** | 1.283 | 0.435 | 1.304 | 0.360 | 1.015 | 0.035 | Increased |
| **GP1BA** | 3.914 | 0.688 | 4.030 | 0.745 | 1.084 | 0.036 | Increased |
| **LILRB2** | 2.854 | 0.339 | 2.900 | 0.339 | 1.032 | 0.039 | Increased |
| **ICAM1** | 5.625 | 0.366 | 5.693 | 0.374 | 1.048 | 0.041 | Increased |
| **PRCP** | 0.900 | 0.319 | 0.972 | 0.388 | 1.051 | 0.045 | Increased |
| **THPO** | 1.713 | 0.317 | 1.762 | 0.323 | 1.034 | 0.045 | Increased |
| **ST1A1** | 3.151 | 1.264 | 3.361 | 1.448 | 1.157 | 0.048 | Increased |
| **GT** | 1.654 | 0.735 | 1.538 | 0.643 | 0.923 | 0.049 | Decreased |
| IL20RA | 0.702 | 0.411 | 0.726 | 0.353 | 1.017 | 0.052 | Increased |
| DCN | 3.949 | 0.254 | 3.914 | 0.277 | 0.976 | 0.052 | Decreased |
| TGFBR3 | 1.098 | 0.306 | 1.157 | 0.362 | 1.042 | 0.060 | Increased |
| SOD1 | 1.130 | 0.467 | 1.207 | 0.549 | 1.055 | 0.066 | Increased |
| Flt3L | 9.178 | 0.411 | 9.225 | 0.446 | 1.032 | 0.070 | Increased |
| ALCAM | 5.037 | 0.248 | 5.078 | 0.269 | 1.029 | 0.071 | Increased |
| REG3A | 0.756 | 0.211 | 0.786 | 0.222 | 1.021 | 0.073 | Increased |
| CST3 | 6.593 | 0.378 | 6.645 | 0.381 | 1.037 | 0.077 | Increased |
| IL4 | 0.061 | 0.538 | 0.093 | 0.471 | 1.022 | 0.077 | Increased |
| CHL1 | 2.545 | 0.326 | 2.595 | 0.333 | 1.035 | 0.078 | Increased |
| FCN2 | 5.147 | 0.719 | 5.218 | 0.636 | 1.051 | 0.079 | Increased |
| NID1 | 2.778 | 0.378 | 2.837 | 0.439 | 1.042 | 0.081 | Increased |
| 4EBP1 | 5.145 | 1.387 | 5.338 | 1.476 | 1.142 | 0.085 | Increased |
| ANG | 7.034 | 0.395 | 7.085 | 0.375 | 1.036 | 0.086 | Increased |
| Notch3 | 4.018 | 0.362 | 3.982 | 0.365 | 0.975 | 0.086 | Decreased |
| GDF15 | 4.998 | 0.396 | 5.067 | 0.438 | 1.049 | 0.087 | Increased |
| CST5 | 6.316 | 0.530 | 6.239 | 0.485 | 0.948 | 0.087 | Decreased |
| HAOX1 | 2.385 | 1.123 | 2.575 | 1.239 | 1.140 | 0.089 | Increased |
| LILRB1 | 0.538 | 0.248 | 0.567 | 0.250 | 1.020 | 0.095 | Increased |
| FETUB | 0.655 | 0.383 | 0.709 | 0.396 | 1.038 | 0.097 | Increased |
| CD4 | 3.790 | 0.418 | 3.847 | 0.411 | 1.040 | 0.098 | Increased |
| SORT1 | 7.716 | 0.367 | 7.758 | 0.363 | 1.029 | 0.099 | Increased |
| ADM | 4.531 | 2.000 | 4.754 | 2.011 | 1.167 | 0.100 | Increased |
| MPO | 3.343 | 0.408 | 3.395 | 0.408 | 1.037 | 0.101 | Increased |
| CHIT1 | 6.338 | 1.416 | 6.087 | 1.624 | 0.840 | 0.105 | Decreased |
| PGF | 7.086 | 0.313 | 7.125 | 0.326 | 1.028 | 0.111 | Increased |
| tPA | 6.754 | 0.641 | 6.830 | 0.582 | 1.054 | 0.112 | Increased |
| COL18A1 | 2.210 | 0.338 | 2.257 | 0.361 | 1.033 | 0.117 | Increased |
| MMP3 | 5.755 | 0.693 | 5.684 | 0.671 | 0.952 | 0.117 | Decreased |
| MMP2 | 2.936 | 0.344 | 2.904 | 0.364 | 0.978 | 0.118 | Decreased |
| FS | 10.364 | 0.599 | 10.443 | 0.653 | 1.057 | 0.118 | Increased |
| TIE2 | 7.982 | 0.291 | 7.950 | 0.265 | 0.978 | 0.121 | Decreased |
| IL7 | 2.368 | 0.837 | 2.470 | 0.810 | 1.073 | 0.122 | Increased |
| TRAIL | 7.186 | 0.416 | 7.243 | 0.407 | 1.041 | 0.123 | Increased |
| LIFR | 2.669 | 0.271 | 2.629 | 0.239 | 0.973 | 0.123 | Decreased |
| FAS | 5.008 | 0.330 | 5.046 | 0.337 | 1.027 | 0.124 | Increased |
| TIMP1 | 4.868 | 0.306 | 4.901 | 0.320 | 1.023 | 0.124 | Increased |
| IGFBP2 | 7.425 | 0.706 | 7.352 | 0.670 | 0.951 | 0.129 | Decreased |
| MCP1_A | 10.150 | 0.489 | 10.210 | 0.507 | 1.043 | 0.129 | Increased |
| ADA | 1.645 | 0.538 | 1.713 | 0.596 | 1.049 | 0.130 | Increased |
| FCGR3B | 3.311 | 0.509 | 3.262 | 0.533 | 0.967 | 0.133 | Decreased |
| CTSD | 4.242 | 0.404 | 4.288 | 0.429 | 1.033 | 0.136 | Increased |
| OSMR | 1.075 | 0.215 | 1.098 | 0.228 | 1.016 | 0.137 | Increased |
| DECR1 | 5.027 | 1.560 | 5.239 | 1.705 | 1.158 | 0.139 | Increased |
| TIMD4 | 3.004 | 0.450 | 3.051 | 0.458 | 1.033 | 0.140 | Increased |
| MMP1 | 10.920 | 1.391 | 11.082 | 1.431 | 1.119 | 0.140 | Increased |
| CCL23 | 9.830 | 0.440 | 9.880 | 0.445 | 1.035 | 0.141 | Increased |
| TGM2 | 8.564 | 0.554 | 8.492 | 0.614 | 0.952 | 0.143 | Decreased |
| IL2 | 0.817 | 0.363 | 0.844 | 0.273 | 1.019 | 0.143 | Increased |
| DNER | 8.699 | 0.255 | 8.732 | 0.261 | 1.023 | 0.145 | Increased |
| GH | 8.920 | 2.057 | 8.675 | 2.095 | 0.844 | 0.146 | Decreased |
| F11 | 6.631 | 0.318 | 6.668 | 0.291 | 1.026 | 0.147 | Increased |
| vWF | 6.889 | 0.785 | 6.957 | 0.751 | 1.048 | 0.150 | Increased |
| FGF19 | 7.379 | 0.884 | 7.280 | 0.880 | 0.934 | 0.150 | Decreased |
| GDF2 | 8.373 | 0.541 | 8.296 | 0.598 | 0.949 | 0.152 | Decreased |
| SRC | 6.394 | 1.041 | 6.468 | 1.080 | 1.052 | 0.162 | Increased |
| FGF5 | 0.038 | 0.320 | 0.075 | 0.328 | 1.026 | 0.162 | Increased |
| CXCL1_A | 9.772 | 0.988 | 9.915 | 0.950 | 1.105 | 0.162 | Increased |
| CXCL11 | 7.693 | 0.854 | 7.831 | 0.972 | 1.100 | 0.162 | Increased |
| PECAM1 | 4.145 | 0.528 | 4.225 | 0.593 | 1.057 | 0.164 | Increased |
| PSPD | 2.521 | 0.629 | 2.592 | 0.638 | 1.050 | 0.165 | Increased |
| PLC | 6.426 | 0.324 | 6.453 | 0.327 | 1.019 | 0.170 | Increased |
| EPHB4 | 4.499 | 0.273 | 4.527 | 0.252 | 1.020 | 0.171 | Increased |
| PAI | 5.718 | 0.860 | 5.809 | 0.849 | 1.065 | 0.173 | Increased |
| PCOLCE | 5.253 | 0.398 | 5.296 | 0.413 | 1.030 | 0.175 | Increased |
| ENG | 1.393 | 0.296 | 1.408 | 0.243 | 1.010 | 0.175 | Increased |
| TNFR1 | 5.753 | 0.335 | 5.788 | 0.324 | 1.024 | 0.180 | Increased |
| UPAR | 4.385 | 0.388 | 4.441 | 0.416 | 1.040 | 0.183 | Increased |
| CCL16 | 6.075 | 0.563 | 6.133 | 0.537 | 1.041 | 0.188 | Increased |
| PLTP | 1.635 | 0.443 | 1.650 | 0.302 | 1.011 | 0.195 | Increased |
| TFF3 | 4.850 | 0.670 | 4.928 | 0.840 | 1.055 | 0.200 | Increased |
| CXCL6 | 8.055 | 0.907 | 8.163 | 0.942 | 1.077 | 0.201 | Increased |
| TNFRSF14 | 4.209 | 0.326 | 4.242 | 0.321 | 1.023 | 0.202 | Increased |
| MMP10 | 5.703 | 0.658 | 5.778 | 0.671 | 1.053 | 0.205 | Increased |
| IL8 | 5.587 | 0.762 | 5.642 | 0.722 | 1.039 | 0.207 | Increased |
| TCN2 | 2.631 | 0.430 | 2.678 | 0.410 | 1.033 | 0.209 | Increased |
| Gal3 | 5.562 | 0.337 | 5.594 | 0.338 | 1.022 | 0.209 | Increased |
| LAPTGFbeta1 | 6.234 | 0.614 | 6.312 | 0.636 | 1.055 | 0.211 | Increased |
| IL2RA | 3.731 | 0.443 | 3.785 | 0.417 | 1.038 | 0.212 | Increased |
| BMP6 | 2.458 | 0.783 | 2.564 | 0.869 | 1.076 | 0.213 | Increased |
| AZU1 | 3.510 | 0.855 | 3.580 | 0.871 | 1.050 | 0.214 | Increased |
| NEMO | 3.134 | 0.882 | 3.243 | 0.955 | 1.079 | 0.214 | Increased |
| MFAP5 | 1.158 | 0.310 | 1.171 | 0.257 | 1.009 | 0.215 | Increased |
| PRSS2 | 1.688 | 0.446 | 1.718 | 0.408 | 1.021 | 0.220 | Increased |
| CCL20 | 5.503 | 1.029 | 5.564 | 0.952 | 1.044 | 0.222 | Increased |
| TNFR2 | 4.894 | 0.333 | 4.927 | 0.331 | 1.024 | 0.224 | Increased |
| CXCL1 | 8.625 | 0.994 | 8.754 | 0.962 | 1.094 | 0.225 | Increased |
| TNFRSF11A | 4.822 | 0.407 | 4.860 | 0.407 | 1.027 | 0.227 | Increased |
| MEGF9 | 1.823 | 0.295 | 1.851 | 0.302 | 1.019 | 0.231 | Increased |
| F7 | 3.692 | 0.331 | 3.731 | 0.370 | 1.027 | 0.239 | Increased |
| TNXB | 0.612 | 0.183 | 0.626 | 0.196 | 1.010 | 0.242 | Increased |
| CCL19 | 8.959 | 0.939 | 9.052 | 1.009 | 1.067 | 0.253 | Increased |
| COMP | 6.444 | 0.439 | 6.416 | 0.463 | 0.981 | 0.253 | Decreased |
| ENRAGE | 2.688 | 0.821 | 2.616 | 0.784 | 0.952 | 0.254 | Decreased |
| CD40L | 3.907 | 1.258 | 4.048 | 1.335 | 1.103 | 0.257 | Increased |
| EFEMP1 | 2.313 | 0.617 | 2.363 | 0.643 | 1.035 | 0.263 | Increased |
| SPON2 | 9.401 | 0.306 | 9.418 | 0.290 | 1.012 | 0.264 | Increased |
| AMBP | 7.226 | 0.244 | 7.239 | 0.222 | 1.009 | 0.272 | Increased |
| BetaNGF | 0.367 | 0.411 | 0.402 | 0.486 | 1.025 | 0.273 | Increased |
| IGLC2 | 6.000 | 0.504 | 6.043 | 0.470 | 1.030 | 0.274 | Increased |
| ICAM3 | 1.902 | 0.327 | 1.867 | 0.279 | 0.976 | 0.276 | Decreased |
| BLMhydrolase | 5.246 | 0.415 | 5.290 | 0.442 | 1.031 | 0.278 | Increased |
| GLO1 | 3.862 | 0.845 | 3.930 | 0.927 | 1.048 | 0.280 | Increased |
| TSLP | 1.233 | 0.641 | 1.255 | 0.588 | 1.016 | 0.283 | Increased |
| IL33 | 0.604 | 0.390 | 0.624 | 0.288 | 1.014 | 0.287 | Increased |
| CCL3 | 5.478 | 0.740 | 5.532 | 0.761 | 1.038 | 0.291 | Increased |
| DEFA1 | 2.109 | 0.362 | 2.124 | 0.282 | 1.011 | 0.293 | Increased |
| TNFRSF13B | 8.301 | 0.478 | 8.309 | 0.373 | 1.006 | 0.293 | Increased |
| SELL | 7.219 | 0.347 | 7.240 | 0.336 | 1.014 | 0.296 | Increased |
| PAR1 | 6.817 | 0.742 | 6.894 | 0.713 | 1.055 | 0.297 | Increased |
| THBS2 | 5.120 | 0.215 | 5.143 | 0.198 | 1.016 | 0.299 | Increased |
| ANGPTL3 | 2.724 | 0.469 | 2.753 | 0.402 | 1.020 | 0.301 | Increased |
| OSM | 5.032 | 0.954 | 5.087 | 0.938 | 1.039 | 0.302 | Increased |
| LTBR | 3.513 | 0.296 | 3.536 | 0.274 | 1.016 | 0.303 | Increased |
| OPG_A | 9.721 | 0.321 | 9.757 | 0.349 | 1.025 | 0.304 | Increased |
| CX3CL1 | 5.875 | 0.452 | 5.896 | 0.424 | 1.015 | 0.319 | Increased |
| PSGL1 | 4.025 | 0.274 | 4.046 | 0.272 | 1.015 | 0.319 | Increased |
| SAA4 | 4.524 | 0.751 | 4.588 | 0.775 | 1.046 | 0.324 | Increased |
| TLT2 | 4.962 | 0.390 | 4.992 | 0.395 | 1.021 | 0.326 | Increased |
| CXCL5 | 10.709 | 1.374 | 10.863 | 1.293 | 1.113 | 0.331 | Increased |
| IL18R1 | 7.170 | 0.421 | 7.201 | 0.403 | 1.022 | 0.332 | Increased |
| GP6 | 1.916 | 0.668 | 1.988 | 0.726 | 1.051 | 0.333 | Increased |
| LPL | 7.268 | 0.839 | 7.333 | 0.830 | 1.046 | 0.335 | Increased |
| CNTN1 | 3.877 | 0.328 | 3.853 | 0.320 | 0.984 | 0.352 | Decreased |
| PRSS27 | 8.352 | 0.510 | 8.388 | 0.484 | 1.025 | 0.353 | Increased |
| CPA1 | 4.463 | 0.684 | 4.489 | 0.610 | 1.018 | 0.354 | Increased |
| Gal4 | 2.729 | 0.404 | 2.750 | 0.413 | 1.014 | 0.355 | Increased |
| MB | 6.016 | 0.550 | 5.974 | 0.542 | 0.972 | 0.375 | Decreased |
| IL22RA1 | 2.161 | 0.561 | 2.184 | 0.544 | 1.016 | 0.381 | Increased |
| hOSCAR | 9.648 | 0.283 | 9.671 | 0.257 | 1.016 | 0.383 | Increased |
| GRN | 5.837 | 0.304 | 5.866 | 0.326 | 1.020 | 0.385 | Increased |
| CXCL16 | 4.579 | 0.284 | 4.598 | 0.306 | 1.014 | 0.388 | Increased |
| ANG1 | 7.858 | 0.942 | 7.922 | 0.955 | 1.045 | 0.390 | Increased |
| CES1 | 1.176 | 0.579 | 1.194 | 0.514 | 1.012 | 0.392 | Increased |
| SERPINA12 | 2.889 | 1.286 | 2.825 | 1.264 | 0.956 | 0.397 | Decreased |
| CA5A | 2.217 | 0.730 | 2.259 | 0.699 | 1.030 | 0.403 | Increased |
| IL1RT1 | 6.007 | 0.274 | 5.998 | 0.276 | 0.994 | 0.406 | Decreased |
| PTPRS | 0.553 | 0.219 | 0.575 | 0.206 | 1.016 | 0.406 | Increased |
| FAP | 1.215 | 0.264 | 1.222 | 0.240 | 1.005 | 0.409 | Increased |
| CCL17 | 8.151 | 0.918 | 8.234 | 0.961 | 1.059 | 0.409 | Increased |
| CRTAC1 | 1.032 | 0.486 | 0.999 | 0.451 | 0.977 | 0.414 | Decreased |
| IL18BP | 5.675 | 0.348 | 5.707 | 0.348 | 1.022 | 0.414 | Increased |
| MBL2 | 8.216 | 1.325 | 8.148 | 1.310 | 0.954 | 0.418 | Decreased |
| NOTCH1 | 2.154 | 0.226 | 2.165 | 0.218 | 1.007 | 0.420 | Increased |
| CPB1 | 5.036 | 0.637 | 5.071 | 0.585 | 1.025 | 0.423 | Increased |
| CD93 | 10.709 | 0.352 | 10.731 | 0.357 | 1.015 | 0.431 | Increased |
| CFHR5 | 7.579 | 0.414 | 7.602 | 0.477 | 1.016 | 0.433 | Increased |
| TNF | 1.005 | 0.510 | 1.031 | 0.498 | 1.019 | 0.434 | Increased |
| CDH1 | 3.707 | 0.394 | 3.739 | 0.409 | 1.023 | 0.444 | Increased |
| LILRB5 | 3.308 | 0.784 | 3.376 | 0.748 | 1.048 | 0.444 | Increased |
| DPP4 | 5.032 | 0.363 | 5.046 | 0.354 | 1.010 | 0.447 | Increased |
| TNFB | 4.072 | 0.461 | 4.067 | 0.468 | 0.996 | 0.449 | Decreased |
| NTproBNP | 1.615 | 0.784 | 1.573 | 0.790 | 0.972 | 0.451 | Decreased |
| PI3 | 3.450 | 1.004 | 3.512 | 0.534 | 1.044 | 0.459 | Increased |
| CCL25 | 5.193 | 0.690 | 5.168 | 0.680 | 0.983 | 0.459 | Decreased |
| TIMP4 | 2.716 | 0.585 | 2.714 | 0.474 | 0.999 | 0.467 | Decreased |
| CASP8 | 5.225 | 1.024 | 5.286 | 1.054 | 1.044 | 0.471 | Increased |
| RAGE | 4.451 | 0.336 | 4.470 | 0.349 | 1.013 | 0.473 | Increased |
| GDNF | 1.630 | 0.460 | 1.594 | 0.393 | 0.975 | 0.486 | Decreased |
| MCP2 | 8.241 | 0.670 | 8.274 | 0.735 | 1.023 | 0.487 | Increased |
| GAS6 | 3.354 | 0.454 | 3.382 | 0.482 | 1.020 | 0.499 | Increased |
| CDH5 | 3.864 | 0.452 | 3.868 | 0.404 | 1.003 | 0.500 | Increased |
| NRP1 | 1.157 | 0.149 | 1.149 | 0.156 | 0.994 | 0.502 | Decreased |
| IL10RA | 0.542 | 0.683 | 0.557 | 0.743 | 1.010 | 0.504 | Increased |
| uPA_A | 9.042 | 0.317 | 9.057 | 0.353 | 1.010 | 0.505 | Increased |
| GNLY | 0.844 | 0.360 | 0.852 | 0.300 | 1.005 | 0.506 | Increased |
| TRAP | 1.705 | 0.410 | 1.745 | 0.442 | 1.028 | 0.508 | Increased |
| IL5 | 1.405 | 1.089 | 1.461 | 1.129 | 1.039 | 0.508 | Increased |
| CDCP1 | 3.058 | 0.587 | 3.091 | 0.602 | 1.023 | 0.513 | Increased |
| PARP1 | 2.313 | 0.985 | 2.369 | 0.994 | 1.040 | 0.518 | Increased |
| KLK6 | 5.191 | 0.329 | 5.174 | 0.343 | 0.988 | 0.524 | Decreased |
| KIM1 | 6.891 | 0.682 | 6.925 | 0.710 | 1.024 | 0.524 | Increased |
| PLXNB2 | 0.816 | 0.245 | 0.807 | 0.231 | 0.994 | 0.535 | Decreased |
| MMP7 | 4.428 | 1.710 | 4.527 | 1.665 | 1.071 | 0.538 | Increased |
| TRANCE | 4.177 | 0.584 | 4.205 | 0.593 | 1.020 | 0.541 | Increased |
| QPCT | 1.624 | 0.287 | 1.659 | 0.330 | 1.025 | 0.543 | Increased |
| XCL1 | 3.878 | 0.781 | 3.931 | 0.761 | 1.037 | 0.550 | Increased |
| VASN | 1.167 | 0.260 | 1.179 | 0.253 | 1.008 | 0.550 | Increased |
| IL1RT2 | 5.801 | 0.306 | 5.815 | 0.315 | 1.010 | 0.553 | Increased |
| PON3 | 4.957 | 0.780 | 4.903 | 0.828 | 0.963 | 0.557 | Decreased |
| AXL | 8.385 | 0.325 | 8.390 | 0.338 | 1.004 | 0.560 | Increased |
| IL1ra | 4.056 | 0.825 | 4.097 | 0.808 | 1.029 | 0.566 | Increased |
| CA1 | 5.801 | 0.844 | 5.771 | 0.862 | 0.980 | 0.566 | Decreased |
| IL20 | 0.130 | 0.480 | 0.121 | 0.377 | 0.994 | 0.568 | Decreased |
| LDLreceptor | 4.357 | 0.581 | 4.386 | 0.627 | 1.020 | 0.569 | Increased |
| SELE | 12.200 | 0.631 | 12.223 | 0.620 | 1.016 | 0.570 | Increased |
| TR | 5.789 | 0.449 | 5.814 | 0.431 | 1.017 | 0.582 | Increased |
| CTSL1 | 6.596 | 0.545 | 6.606 | 0.520 | 1.007 | 0.582 | Increased |
| IL12B | 4.351 | 0.597 | 4.375 | 0.577 | 1.017 | 0.595 | Increased |
| TF | 5.554 | 0.336 | 5.567 | 0.307 | 1.009 | 0.604 | Increased |
| CNDP1 | 2.394 | 0.665 | 2.376 | 0.650 | 0.987 | 0.604 | Decreased |
| SHPS1 | 2.909 | 0.350 | 2.924 | 0.390 | 1.010 | 0.607 | Increased |
| TFPI | 9.182 | 0.347 | 9.195 | 0.332 | 1.009 | 0.608 | Increased |
| IL16 | 5.349 | 0.700 | 5.352 | 0.667 | 1.002 | 0.613 | Increased |
| NRTN | 0.415 | 0.451 | 0.427 | 0.493 | 1.008 | 0.614 | Increased |
| CA4 | 1.166 | 0.281 | 1.152 | 0.252 | 0.991 | 0.616 | Decreased |
| EpCAM | 5.789 | 1.011 | 5.744 | 1.041 | 0.970 | 0.622 | Decreased |
| ST6GAL1 | 2.777 | 0.369 | 2.786 | 0.359 | 1.006 | 0.639 | Increased |
| IL13 | 1.246 | 0.473 | 1.260 | 0.498 | 1.010 | 0.645 | Increased |
| ST2 | 3.699 | 0.469 | 3.693 | 0.453 | 0.996 | 0.656 | Decreased |
| CD84 | 4.362 | 0.529 | 4.355 | 0.548 | 0.995 | 0.658 | Decreased |
| SLAMF1 | 2.120 | 0.561 | 2.132 | 0.463 | 1.009 | 0.662 | Increased |
| LYVE1 | 4.084 | 0.336 | 4.095 | 0.323 | 1.008 | 0.665 | Increased |
| CCL4 | 5.795 | 0.715 | 5.824 | 0.706 | 1.020 | 0.667 | Increased |
| OPN | 4.545 | 0.718 | 4.544 | 0.703 | 0.999 | 0.670 | Decreased |
| CCL15 | 7.026 | 0.421 | 7.037 | 0.407 | 1.007 | 0.672 | Increased |
| C2 | 4.651 | 0.701 | 4.679 | 0.673 | 1.020 | 0.681 | Increased |
| TNFSF14 | 3.815 | 0.770 | 3.831 | 0.702 | 1.011 | 0.688 | Increased |
| MCP4 | 13.917 | 0.665 | 13.961 | 0.723 | 1.030 | 0.691 | Increased |
| PLA2G7 | 1.226 | 0.356 | 1.242 | 0.329 | 1.011 | 0.695 | Increased |
| NCAM1 | 2.457 | 0.267 | 2.455 | 0.277 | 0.998 | 0.697 | Decreased |
| VSIG2 | 2.768 | 0.422 | 2.800 | 0.456 | 1.023 | 0.697 | Increased |
| IL17D | 2.013 | 0.312 | 2.016 | 0.288 | 1.002 | 0.709 | Increased |
| IL15RA | 0.633 | 0.246 | 0.619 | 0.208 | 0.990 | 0.709 | Decreased |
| NT3 | 1.216 | 0.506 | 1.203 | 0.423 | 0.991 | 0.712 | Decreased |
| MARCO | 5.466 | 0.392 | 5.447 | 0.428 | 0.987 | 0.717 | Decreased |
| COL1A1 | 3.034 | 0.399 | 3.045 | 0.388 | 1.008 | 0.718 | Increased |
| SLAMF7 | 3.011 | 0.511 | 2.996 | 0.460 | 0.989 | 0.719 | Decreased |
| BNP | 0.343 | 0.210 | 0.361 | 0.240 | 1.013 | 0.719 | Increased |
| MMP9 | 4.687 | 0.816 | 4.697 | 0.799 | 1.007 | 0.720 | Increased |
| PGLYRP1 | 6.914 | 0.655 | 6.893 | 0.628 | 0.985 | 0.720 | Decreased |
| IgGFcreceptorIIb | 2.069 | 0.668 | 2.042 | 0.682 | 0.981 | 0.723 | Decreased |
| IDUA | 3.645 | 0.940 | 3.654 | 0.933 | 1.007 | 0.733 | Increased |
| CCL28 | 1.879 | 0.432 | 1.905 | 0.455 | 1.018 | 0.736 | Increased |
| KIT | 3.312 | 0.353 | 3.302 | 0.387 | 0.993 | 0.737 | Decreased |
| CCL14 | 4.828 | 0.338 | 4.837 | 0.333 | 1.006 | 0.739 | Increased |
| CCL11 | 6.762 | 0.599 | 6.800 | 0.566 | 1.027 | 0.741 | Increased |
| APOM | 5.713 | 0.464 | 5.726 | 0.489 | 1.010 | 0.748 | Increased |
| IL17RA | 3.504 | 0.539 | 3.491 | 0.496 | 0.991 | 0.752 | Decreased |
| REG1A | 5.783 | 0.559 | 5.789 | 0.518 | 1.004 | 0.754 | Increased |
| uPA | 5.034 | 0.371 | 5.029 | 0.399 | 0.996 | 0.764 | Decreased |
| PIgR | 5.726 | 0.230 | 5.736 | 0.225 | 1.007 | 0.777 | Increased |
| MCP1 | 3.401 | 0.466 | 3.411 | 0.417 | 1.007 | 0.787 | Increased |
| TNFRSF10C | 5.716 | 0.491 | 5.722 | 0.501 | 1.004 | 0.788 | Increased |
| IGFBP3 | 4.177 | 0.364 | 4.157 | 0.392 | 0.986 | 0.792 | Decreased |
| PAM | 1.124 | 0.410 | 1.142 | 0.434 | 1.012 | 0.792 | Increased |
| CXCL10 | 9.647 | 0.820 | 9.668 | 0.814 | 1.015 | 0.804 | Increased |
| ITGB2 | 6.478 | 0.364 | 6.481 | 0.349 | 1.003 | 0.805 | Increased |
| APN | 4.744 | 0.328 | 4.742 | 0.309 | 0.998 | 0.807 | Decreased |
| SPARCL1 | 1.173 | 0.274 | 1.174 | 0.267 | 1.001 | 0.815 | Increased |
| HO1 | 10.528 | 0.640 | 10.538 | 0.661 | 1.007 | 0.818 | Increased |
| IL7R | 1.335 | 0.444 | 1.321 | 0.393 | 0.990 | 0.819 | Decreased |
| CCL24 | 5.596 | 0.985 | 5.594 | 0.929 | 0.998 | 0.821 | Decreased |
| C1QTNF1 | 3.210 | 0.535 | 3.257 | 0.680 | 1.033 | 0.828 | Increased |
| IL1RL2 | 3.524 | 0.398 | 3.520 | 0.447 | 0.997 | 0.833 | Decreased |
| PAPPA | 1.788 | 0.373 | 1.799 | 0.380 | 1.008 | 0.837 | Increased |
| CD163 | 7.141 | 0.515 | 7.124 | 0.510 | 0.988 | 0.837 | Decreased |
| VCAM1 | 3.802 | 0.318 | 3.810 | 0.329 | 1.005 | 0.838 | Increased |
| ACE2 | 2.784 | 0.615 | 2.785 | 0.581 | 1.001 | 0.840 | Increased |
| CHI3L1 | 6.337 | 0.828 | 6.356 | 0.832 | 1.014 | 0.852 | Increased |
| TGFBI | 7.545 | 0.405 | 7.557 | 0.417 | 1.008 | 0.853 | Increased |
| CEACAM8 | 3.114 | 0.680 | 3.127 | 0.719 | 1.009 | 0.859 | Increased |
| IL6RA | 12.174 | 0.383 | 12.165 | 0.384 | 0.994 | 0.860 | Decreased |
| MET | 1.824 | 0.219 | 1.819 | 0.216 | 0.997 | 0.861 | Decreased |
| CR2 | 5.070 | 0.475 | 5.065 | 0.494 | 0.996 | 0.861 | Decreased |
| IL18_A | 7.896 | 0.746 | 7.911 | 0.788 | 1.011 | 0.863 | Increased |
| AOC3 | 2.942 | 0.354 | 2.944 | 0.362 | 1.001 | 0.865 | Increased |
| CA3 | -0.233 | 0.278 | -0.235 | 0.313 | 0.999 | 0.867 | Decreased |
| Dkk1 | 8.974 | 0.725 | 9.001 | 0.766 | 1.019 | 0.872 | Increased |
| LCN2 | 1.655 | 0.458 | 1.630 | 0.362 | 0.983 | 0.876 | Decreased |
| PROC | 3.923 | 0.400 | 3.932 | 0.384 | 1.006 | 0.877 | Increased |
| IL2RB | 1.351 | 0.442 | 1.348 | 0.369 | 0.998 | 0.883 | Decreased |
| OPG | 3.505 | 0.305 | 3.519 | 0.337 | 1.010 | 0.886 | Increased |
| PRTN3 | 4.753 | 0.651 | 4.756 | 0.635 | 1.002 | 0.891 | Increased |
| ICAM2 | 4.900 | 0.386 | 4.896 | 0.404 | 0.997 | 0.894 | Decreased |
| IGFBP6 | 3.875 | 0.339 | 3.879 | 0.319 | 1.002 | 0.903 | Increased |
| TNC | 1.564 | 0.452 | 1.564 | 0.451 | 1.000 | 0.904 | Decreased |
| CXCL9 | 7.242 | 0.705 | 7.220 | 0.690 | 0.985 | 0.905 | Decreased |
| TGFalpha | 3.796 | 0.725 | 3.787 | 0.699 | 0.994 | 0.911 | Decreased |
| AGRP | 2.852 | 0.591 | 2.850 | 0.590 | 0.999 | 0.911 | Decreased |
| ITGAM | 0.860 | 0.405 | 0.855 | 0.300 | 0.996 | 0.913 | Decreased |
| PTX3 | 1.999 | 0.422 | 1.991 | 0.388 | 0.994 | 0.914 | Decreased |
| HBEGF | 3.601 | 0.893 | 3.633 | 0.954 | 1.023 | 0.917 | Increased |
| TM | 8.758 | 0.341 | 8.756 | 0.326 | 0.998 | 0.918 | Decreased |
| REN | 7.028 | 0.778 | 7.037 | 0.752 | 1.007 | 0.921 | Increased |
| BOC | 3.577 | 0.325 | 3.580 | 0.327 | 1.002 | 0.923 | Increased |
| THBS4 | 4.114 | 0.567 | 4.118 | 0.555 | 1.003 | 0.927 | Increased |
| FABP2 | 7.464 | 0.760 | 7.483 | 0.728 | 1.013 | 0.928 | Increased |
| GIF | 6.091 | 0.866 | 6.104 | 0.858 | 1.009 | 0.930 | Increased |
| MERTK | 4.642 | 0.383 | 4.640 | 0.403 | 0.998 | 0.932 | Decreased |
| IL4RA | 1.487 | 0.282 | 1.499 | 0.358 | 1.009 | 0.945 | Increased |
| MEPE | 3.779 | 0.679 | 3.787 | 0.707 | 1.006 | 0.948 | Increased |
| UMOD | 0.731 | 0.264 | 0.733 | 0.284 | 1.001 | 0.958 | Increased |
| PDGFsubunitB | 10.154 | 0.814 | 10.182 | 0.776 | 1.020 | 0.959 | Increased |
| LOX1 | 6.695 | 0.857 | 6.679 | 0.847 | 0.989 | 0.961 | Decreased |
| IGFBP7 | 6.746 | 0.347 | 6.753 | 0.354 | 1.005 | 0.961 | Increased |
| RETN | 6.264 | 0.500 | 6.259 | 0.477 | 0.997 | 0.961 | Decreased |
| DLK1 | 5.845 | 0.616 | 5.844 | 0.630 | 0.999 | 0.963 | Decreased |
| PDGFsubunitA | 3.908 | 0.879 | 3.942 | 0.883 | 1.024 | 0.963 | Increased |
| FCGR2A | 1.820 | 0.771 | 1.826 | 0.726 | 1.004 | 0.964 | Increased |
| SERPINA5 | 8.686 | 0.345 | 8.651 | 0.712 | 0.976 | 0.969 | Decreased |
| PDL2 | 2.224 | 0.459 | 2.208 | 0.428 | 0.989 | 0.970 | Decreased |
| IL18 | 7.675 | 0.768 | 7.674 | 0.796 | 1.000 | 0.971 | Decreased |
| EGFR | 2.564 | 0.212 | 2.567 | 0.214 | 1.002 | 0.971 | Increased |
| TIE1 | 1.219 | 0.215 | 1.216 | 0.214 | 0.998 | 0.973 | Decreased |
| CTRC | 9.423 | 0.684 | 9.422 | 0.609 | 0.999 | 0.974 | Decreased |
| PRELP | 5.512 | 0.325 | 5.515 | 0.324 | 1.003 | 0.989 | Increased |
| VEGFD | 6.295 | 0.491 | 6.306 | 0.418 | 1.007 | 0.989 | Increased |
| TWEAK | 8.958 | 0.553 | 8.967 | 0.581 | 1.006 | 0.989 | Increased |
| ADAMTS13 | 5.093 | 0.308 | 5.077 | 0.249 | 0.989 | 0.990 | Decreased |
| LTBP2 | 1.002 | 0.251 | 1.002 | 0.264 | 1.000 | 0.994 | Decreased |
| CD6 | 5.062 | 0.549 | 5.065 | 0.557 | 1.002 | 0.997 | Increased |
| IL1alpha | 0.542 | 0.903 | 0.510 | 0.711 | 0.978 | 0.999 | Decreased |

**Supplemental Table 3- Biological processes identified by gene ontology enrichment analysis to be represented by the 23 proteins that were identified by the machine learning model.**

| Term | GO_BP | Fold Enrichment | p-value | FDR | Proteins |
| --- | --- | --- | --- | --- | --- |
| GO:0006954 | inflammatory response | 15.78 | <0.001 | <0.001 | JAM-A, CSF-1, IL27, CCL18, Gal-9, CCL5, SELP, IL6 |
| GO:0032733 | positive regulation of interleukin-10 production | 80.39 | <0.001 | 0.004 | HGF, Gal-9, PD-L1, IL6 |
| GO:0042102 | positive regulation of T cell proliferation | 50.39 | <0.001 | 0.010 | CCL5, PD-L1, SCF, IL6 |
| GO:0002687 | positive regulation of leukocyte migration | 168.82 | <0.001 | 0.010 | VEGFA, SELP, SCF |
| GO:0046007 | negative regulation of activated T cell proliferation | 158.27 | <0.001 | 0.010 | Gal-9, PD-L1, CASP-3 |
| GO:0030335 | positive regulation of cell migration | 16.11 | <0.001 | 0.012 | HGF, CSF-1, VEGFA, CCL5, PD-L1 |
| GO:0008284 | positive regulation of cell proliferation | 9.36 | <0.001 | 0.016 | CSF-1, FGF-23, VEGFA, IL-24, SCF, IL6 |
| GO:0032496 | response to lipopolysaccharide | 23.61 | 0.001 | 0.027 | CSF-1, Gal-9, SELP, CASP-3 |
| GO:0007159 | leukocyte cell-cell adhesion | 79.13 | 0.001 | 0.027 | JAM-A, CCL5, SELP |
| GO:0002548 | monocyte chemotaxis | 56.27 | 0.001 | 0.049 | CCL18, CCL5, IL6 |
| GO:0050918 | positive chemotaxis | 52.76 | 0.001 | 0.052 | HGF, VEGFA, CCL5 |
| GO:0071346 | cellular response to interferon-gamma | 25.58 | 0.006 | 0.159 | CCL18, Gal-9, CCL5 |
| GO:0006935 | chemotaxis | 20.59 | 0.008 | 0.219 | CCL18, Gal-9, CCL5 |
| GO:0007155 | cell adhesion | 7.50 | 0.003 | 0.108 | GP1BA, JAM-A, SPON1, SELP, SCF |
| GO:0006955 | immune response | 6.79 | 0.018 | 0.305 | CCL18, CCL5, PD-L1, CD8A |
| GO:1903238 | positive regulation of leukocyte tethering or rolling | 129.86 | 0.015 | 0.265 | GP1BA, SELP |
| GO:0032682 | negative regulation of chemokine production | 153.47 | 0.012 | 0.265 | Gal-9, IL6 |
| GO:0030224 | monocyte differentiation | 88.85 | 0.021 | 0.305 | CSF-1, VEGFA |
| GO:0048245 | eosinophil chemotaxis | 60.29 | 0.031 | 0.414 | CCL18, CCL5 |
| GO:0030225 | macrophage differentiation | 56.27 | 0.033 | 0.433 | CSF-1, VEGFA |
| GO:0048247 | lymphocyte chemotaxis | 49.65 | 0.038 | 0.467 | CCL18, CCL5 |
| GO:0010759 | positive regulation of macrophage chemotaxis | 88.85 | 0.021 | 0.305 | CSF-1, CCL5 |
| GO:0030593 | neutrophil chemotaxis | 20.59 | 0.089 | 0.680 | CCL18, CCL5 |
| GO:0034097 | response to cytokine | 35.17 | 0.053 | 0.554 | SELP, PD-L1 |
| GO:0070374 | positive regulation of ERK1 and ERK2 cascade | 18.76 | <0.001 | 0.010 | FGF-23, CCL18, VEGFA, Gal-9, CCL5 |
| GO:0014068 | positive regulation of phosphatidylinositol 3-kinase signaling | 39.26 | <0.001 | 0.010 | HGF, VEGFA, CCL5, SELP |
| GO:0050731 | positive regulation of peptidyl-tyrosine phosphorylation | 37.10 | <0.001 | 0.010 | HGF, VEGFA, SCF, IL6 |
| GO:0048012 | hepatocyte growth factor receptor signaling pathway | 187.57 | 0.010 | 0.251 | HGF, SIRT2 |
| GO:0010572 | positive regulation of platelet activation | 168.82 | 0.011 | 0.265 | GP1BA, SELP |
| GO:0043410 | positive regulation of MAPK cascade | 15.63 | 0.014 | 0.265 | HGF, VEGFA, IL6 |
| GO:1901731 | positive regulation of platelet aggregation | 129.86 | 0.015 | 0.265 | JAM-A, IL6 |
| GO:0035729 | cellular response to hepatocyte growth factor stimulus | 105.51 | 0.018 | 0.305 | HGF, SIRT2 |
| GO:0043547 | positive regulation of GTPase activity | 13.40 | 0.019 | 0.305 | JAM-A, CCL18, CCL5 |
| GO:0001934 | positive regulation of protein phosphorylation | 11.89 | 0.024 | 0.334 | HGF, FGF-23, VEGFA |
| GO:0046579 | positive regulation of Ras protein signal transduction | 73.40 | 0.026 | 0.350 | CSF-1, SCF |
| GO:0042327 | positive regulation of phosphorylation | 44.43 | 0.042 | 0.509 | VEGFA, CCL5 |
| GO:0046427 | positive regulation of JAK-STAT cascade | 37.51 | 0.050 | 0.554 | CCL5, IL6 |
| GO:0032689 | negative regulation of interferon-gamma production | 36.70 | 0.051 | 0.554 | Gal-9, PD-L1 |
| GO:0007166 | cell surface receptor signaling pathway | 7.84 | 0.051 | 0.554 | GP1BA, PD-L1, CD8A |
| GO:0051781 | positive regulation of cell division | 34.45 | 0.054 | 0.554 | SIRT2, VEGFA |
| GO:0043491 | protein kinase B signaling | 31.85 | 0.058 | 0.586 | SIRT2, CCL5 |
| GO:0070371 | ERK1 and ERK2 cascade | 30.69 | 0.061 | 0.586 | FGF-23, Gal-9 |
| GO:0048661 | positive regulation of smooth muscle cell proliferation | 29.11 | 0.064 | 0.586 | CCL5, IL6 |
| GO:0070098 | chemokine-mediated signaling pathway | 24.12 | 0.076 | 0.629 | CCL18, CCL5 |
| GO:0043406 | positive regulation of MAP kinase activity | 21.92 | 0.084 | 0.659 | VEGFA, SCF |
| GO:0045599 | negative regulation of fat cell differentiation | 28.14 | 0.066 | 0.586 | SIRT2, IL6 |
| GO:0050796 | regulation of insulin secretion | 36.70 | 0.051 | 0.554 | CCL5, IL6 |
| GO:0071374 | cellular response to parathyroid hormone stimulus | 153.47 | 0.012 | 0.265 | CSF-1, FGF-23 |
| GO:0051384 | response to glucocorticoid | 29.62 | 0.063 | 0.586 | IL6, CASP-3 |
| GO:0016485 | protein processing | 19.86 | 0.092 | 0.685 | SPON1, CASP-3 |
