# Supplementary figures and images for: High dietary inflammatory index associates with inflammatory proteins in plasma
Source: Diabetol Metab Syndr. 2024 Feb 26;16:50. doi: 10.1186/s13098-024-01287-y (PMC10895728; doi:10.1186/s13098-024-01287-y)

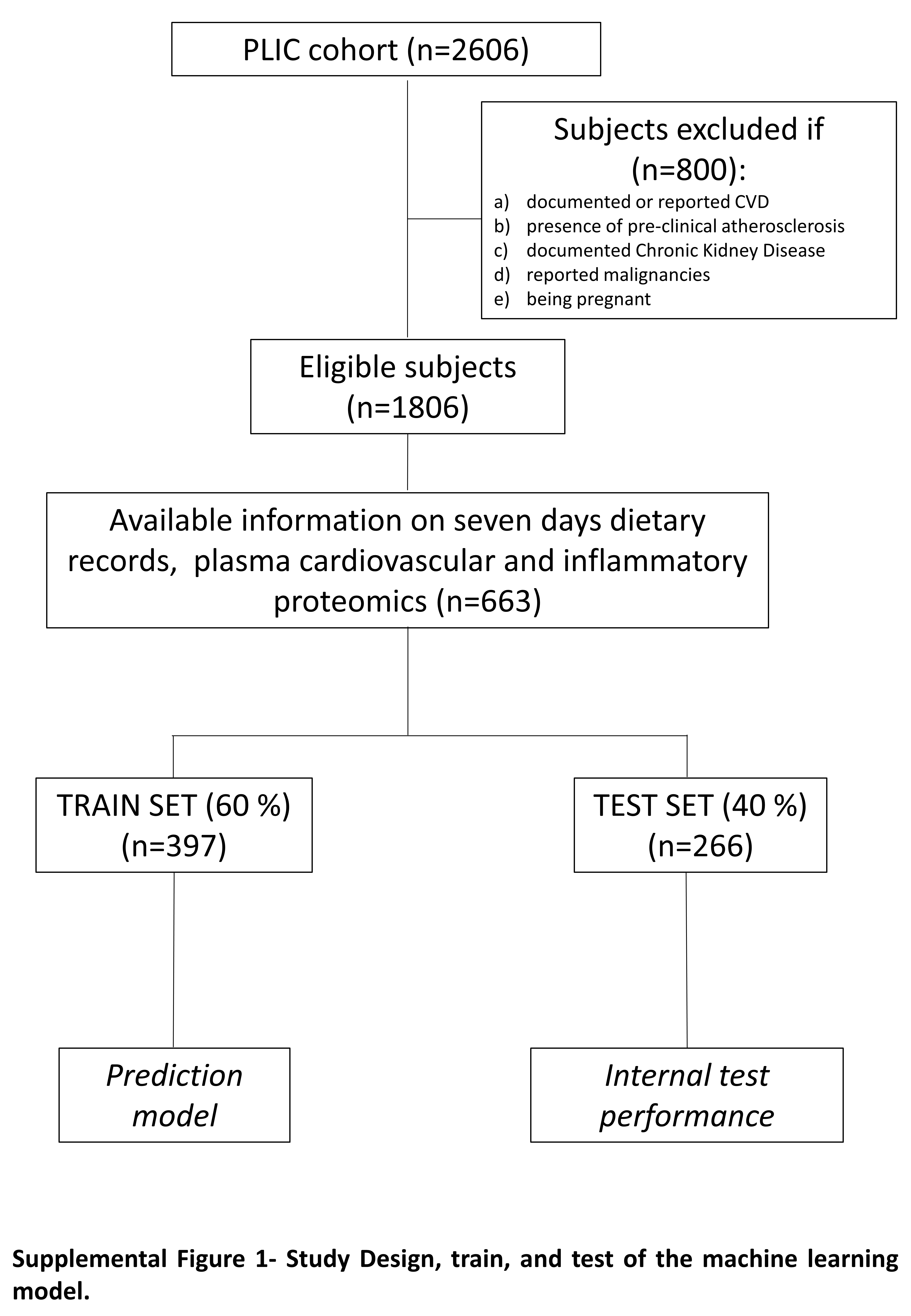

Supplement: Supplementary file 2 — Supplementary Material 2 [file 13098_2024_1287_MOESM2_ESM.png]

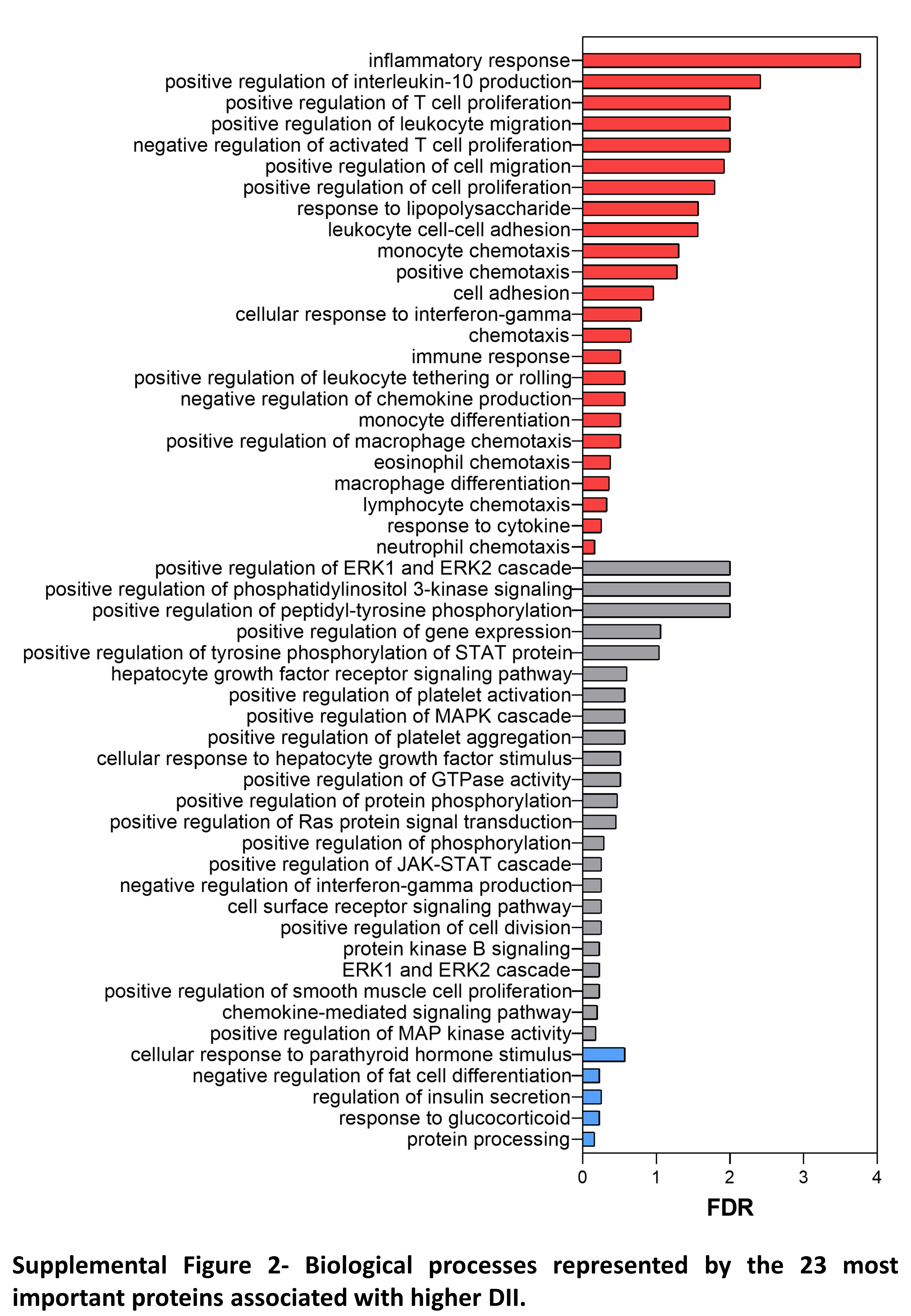

Supplement: Supplementary file 3 — Supplementary Material 3 [file 13098_2024_1287_MOESM3_ESM.png]
